# Transcriptomic analysis of human ALS skeletal muscle reveals a disease-specific pattern of dysregulated circRNAs

**DOI:** 10.18632/aging.204450

**Published:** 2022-12-30

**Authors:** Dimitrios Tsitsipatis, Krystyna Mazan-Mamczarz, Ying Si, Allison B. Herman, Jen-Hao Yang, Abhishek Guha, Yulan Piao, Jinshui Fan, Jennifer L. Martindale, Rachel Munk, Xiaoling Yang, Supriyo De, Brijesh K. Singh, Ritchie Ho, Myriam Gorospe, Peter H. King

**Affiliations:** 1 Laboratory of Genetics and Genomics, National Institute on Aging, Intramural Research Program, National Institutes of Health, Baltimore, MD 21224, USA; 2 Department of Neurology, The University of Alabama at Birmingham, Birmingham, AL 35294, USA; 3 Birmingham Veterans Affairs Medical Center, Birmingham, AL 35294, USA; 4 Center for Neural Science and Medicine, Cedars-Sinai Medical Center, Los Angeles, CA 90048, USA; 5 Department of Biomedical Sciences, Cedars-Sinai Medical Center, Los Angeles, CA 90048, USA; 6 Board of Governors Regenerative Medicine Institute, Cedars-Sinai Medical Center, Los Angeles, CA 90048, USA; 7 Department of Neurology, Cedars-Sinai Medical Center, Los Angeles, CA 90048, USA; 8 Center for Neurodegeneration and Experimental Therapeutics, The University of Alabama at Birmingham, Birmingham, AL 35294, USA; 9 Cell, Developmental, and Integrative Biology, The University of Alabama at Birmingham, Birmingham, AL 35294, USA

**Keywords:** amyotrophic lateral sclerosis, circular RNAs, neurodegenerative disease, human skeletal muscle, human spinal cord tissue

## Abstract

Circular RNAs are abundant, covalently closed transcripts that arise in cells through back-splicing and display distinct expression patterns across cells and developmental stages. While their functions are largely unknown, their intrinsic stability has made them valuable biomarkers in many diseases. Here, we set out to examine circRNA patterns in amyotrophic lateral sclerosis (ALS). By RNA-sequencing analysis, we first identified circRNAs and linear RNAs that were differentially abundant in skeletal muscle biopsies from ALS compared to normal individuals. By RT-qPCR analysis, we confirmed that 8 circRNAs were significantly elevated and 10 were significantly reduced in ALS, while the linear mRNA counterparts, arising from shared precursor RNAs, generally did not change. Several of these circRNAs were also differentially abundant in motor neurons derived from human induced pluripotent stem cells (iPSCs) bearing ALS mutations, and across different disease stages in skeletal muscle from a mouse model of ALS (SOD1^G93A^). Interestingly, a subset of the circRNAs significantly elevated in ALS muscle biopsies were significantly reduced in the spinal cord samples from ALS patients and ALS (SOD1^G93A^) mice. In sum, we have identified differentially abundant circRNAs in ALS-relevant tissues (muscle and spinal cord) that could inform about neuromuscular molecular programs in ALS and guide the development of therapies.

## INTRODUCTION

Amyotrophic lateral sclerosis (ALS) is a progressive and fatal neurodegenerative disease of motor neurons. Aging is a major risk factor for ALS, and the median length of survival after symptom onset is typically ~3–5 years [1, 2]. Over 30 genes have been identified as triggers for familial ALS, comprising ~10% of cases, whereas other gene mutations and/or environmental triggers likely underlie sporadic ALS (~90% of cases) [1, 3–5]. Clinically, patients present with a wide range of signs and symptoms related to a combination of upper and lower motor neuron dysfunction [6, 7].

This extensive clinical and genetic heterogeneity suggests that ALS is a syndrome rather than a specific disease [1]. However, once triggered, motor neuron degeneration in classical ALS often follows a predictable pattern, suggesting the existence of common molecular pathways of progression [6].

Skeletal muscle and the neuromuscular junction (NMJ) are critical end points of the motor neuron system and reflect the earliest manifestations of disease pathology, which include mitochondrial dysfunction, NMJ destruction, atrophy, and distal axonopathy [8–12]. In normal states, there is robust communication between muscle fibers and motor neurons through, for example, the release of growth factors neurotrophin-4 (NT4) and insulin-like growth factor 1 (IGF1) by the muscle and the release of acetylcholine by neurons [13–15]. Thus, aberrant communication at the NMJ may represent a common pathway in ALS disease progression independent of the initial trigger. In support of this hypothesis, we have identified an extensive program of altered molecular markers in skeletal muscle, including transforming growth factor beta 1 (TGF-β1) and mothers against decapentaplegic homolog 8 (SMAD8), that are consistently increased across a broad sampling of ALS patients [16–21]. The similarity of this pattern in skeletal muscle of the superoxide dismutase 1 G93A mutant (SOD1^G93A^) mouse further underscores this hypothesis, as mutations in the *SOD1* gene locus represent only ~2% of all ALS [22]. These markers are also detected in early presymptomatic stages in the SOD1^G93A^ mouse and increase as the disease advances, suggesting their possible usefulness to track disease progression. Reductions in the expression of noncoding RNAs such as muscle microRNAs miR-1, miR-133a, and miR-133b, are also part of this molecular pattern [17], and we have recently found a regulatory link with SMAD8 that suggests that these biomarkers are functionally connected [23]. This evidence prompted us to look for other regulatory molecules, such as circular (circ)RNAs, that might be orchestrating this ALS-specific molecular program in skeletal muscle.

CircRNAs are covalently closed long noncoding (lnc)RNAs which are typically generated during splicing of precursor RNAs, but instead of forming a linear mature transcript, the ends of a segment of the precursor RNA are relegated to form a loop [24–26]. Although circRNAs were originally thought to be byproducts of back-splicing with no distinct roles in biological processes, evidence is now accumulating that circRNAs are key transcriptional, post-transcriptional, and post-translational regulators of gene expression programs that influence cellular responses and function [27–29]. Some abundant circRNAs can associate with microRNAs through regions of complementarity and ‘sponge’ the microRNAs away from target mRNAs and enable translation of the mRNAs. An example in the area of neurodegeneration is *circFgfr2*, which promotes myogenesis by binding miR-133, and in turn enables the activation of the c-Jun N-terminal kinase/mitogen-activated protein kinase (JNK/MAPK) pathway [30]. Other abundant circRNAs can play key roles in muscle differentiation and regeneration [31–33]; for example, *circSamd4* can sequester purine-rich binding proteins (PUR) alpha and beta (PURA, PURB), thus preventing their interaction with the myosin heavy chain (*MHC*) promoter and enhancing myogenesis [34].

In addition to their physiological roles in skeletal muscle, circRNAs are being recognized as potential biomarkers in diseases of the nervous system, including ALS, Alzheimer’s disease, and other non-neurological diseases like lupus, diabetes, and glioblastoma [27, 35]. Recently, *circSMOX* RNA was identified as a biomarker in the SOD1^G93A^ mouse with potential for tracking disease progression and other clinical features [36]. The importance of circRNAs in muscle development and regeneration as well as their potential as biomarkers in ALS prompted us to look systematically at the muscle circRNA transcriptome in patients with ALS. Validation studies of identified circRNAs were extended to other levels of the motor system in post-mortem ALS tissues and to similar tissues in the SOD1^G93A^ mouse. Our long-term goal is to understand their role in the molecular program that is broadly activated in the neuromuscular system of ALS patients and their potential as novel therapeutic targets in ALS.

## MATERIALS AND METHODS

### Human sample collection

Spinal cord and frontal cortex tissue samples were harvested post-mortem from patients enrolled in an ALS tissue collection program (directed by Peter H. King) approved by the University of Alabama at Birmingham (UAB) Institutional Review Board (IRB-100908007 and IRB-091222037). Muscle biopsy samples were obtained from an archive of tissue remnants in the UAB Neuromuscular Division. Neuropathy and myopathy samples were chosen based on histological evidence of denervation or myopathic changes, as determined by a neuromuscular pathologist. All samples were stored at −80°C until use. All patients had a definite diagnosis of ALS based on revised El Escorial criteria.

### Mouse sample collection

All animal protocols were approved by the Institutional Animal Care and Use Committee at UAB in compliance with the National Research Council Guide for the Care and Use of Laboratory Animals. B6.Cg-Tg (SOD1^G93A^) 1 Gur/J mice (the Jackson Laboratory, ME) were mated with C57BL/6J to generate hemizygous SOD1^G93A^ offspring. Age-matched wild-type (WT) littermates were used as controls. Clinical progression was evaluated as described previously [18]. Tissue samples were collected from SOD1^G93A^ and WT littermate controls at post-natal days 60, 125, and 150 as previously described [18].

### Motor neuron differentiation

Human induced pluripotent stem cell (iPSC) lines were maintained at 70% confluency in supplemented media purchased from STEMCELL Technologies [37]. To begin iPSCs differentiation, cells were dissociated into single cells with ReLeSR and then seeded into Matrigel-coated 6-well plates. The cells were then treated with Iscove’s modified Dulbecco’s medium (IMDM) supplemented with B27–vitamin A (2%), N2 (1%), SB431542, CHIR99021, and LDN193189 for 6 days to force them towards a neuroectodermal fate. Fifty percent of the culture medium was replenished every other day. To push cells toward neural progenitors, the cells were gently dissociated into single cells by accutase treatment on day 6 and grown in neural differentiation media supplemented with caudalizing factors all-trans retinoic acid (Sigma R2625) and smoothened agonist (SAG; Cayman 11914) for the next 6 days. Starting at day 12, caudalized neural progenitor cells (NPCs) were maintained in IMDM containing neurogenic small molecules brain-derived neurotrophic factor (BDNF; PeproTech) and glial cell-derived neurotrophic factor (GDNF; Peprotech) for the next 20 days which differentiated NPCs into motor neurons. At day 32, cells were utilized in downstream analyses.

### RNA isolation

For muscle tissues, samples were homogenized in TRIzol reagent (Thermo Fisher Scientific). Chloroform was added to the lysate, and then thoroughly mixed by vortexing. After centrifugation of muscle sample lysates, the aqueous phase was transferred to an RNAspin Mini Column (GE Healthcare) to isolate total RNA following the manufacturer’s protocol. The cultured iPSC-derived motor neurons were harvested and lysed in TRIzol followed by mixing with chloroform, then a Qiagen RNeasy mini kit was used for the following steps. Briefly, after centrifugation, the aqueous phase containing RNA was transferred to a new microfuge tube, and the RNA was precipitated with ethanol. Precipitated RNA was dissolved, passed through the RNeasy column, and treated with DNase to remove genomic DNA contamination. The RNA was eluted in RNase/DNase-free water and quantified using a Nanodrop instrument.

### circRNA enrichment, library preparation, and sequencing

RNA quality and quantity were assessed using the Agilent RNA 6000 nano kit in 2100 Bioanalyzer System (Agilent). High-quality RNA (500 ng) was used to prepare the sequencing library with an Illumina TruSeq Stranded Total RNA Library prep kit following the manufacturer’s protocol. Briefly, after rRNA depletion and cDNA generation, the cDNAs were subjected to 3’ end adenylation, adapter ligation, and purification with AMPure beads (Beckman Coulter). The products were size-selected with SPRIselect beads (Beckman Coulter), and the selected cDNAs were enriched by PCR and repurified with SPRIselect beads to generate the final libraries. The quality and quantity of sequencing libraries were checked using Agilent DNA 1000 Screen Tape on the Agilent TapeStation. Single-read sequencing was performed for 124 cycles with an Illumina NovaSeq 6000 sequencer.

CircRNA was enriched as previously described [38] with minor modifications. Briefly, 2 μg of RNA were treated with 20 U of RNase R (RNR07250, Epicentre) for 1 h at 37°C, after which RNA was isolated using Direct-zol RNA kit (Zymo Research) following the manufacturer’s instructions; the isolated RNA was polyadenylated using a poly(A) tailing kit (Invitrogen) and then selected using Oligo(dT)_25_ Dynabeads (Invitrogen) following the manufacturer’s instructions. From the supernatant, which contained the enriched circRNA pool, RNA was isolated using Direct-zol RNA kit (Zymo Research) following the manufacturer’s instructions; libraries were generated using the entire material, omitting the rRNA depletion steps, and quantified as described above. Paired-end sequencing was performed for 105 cycles with an Illumina HiSeq 2500 sequencer.

### Reverse transcription (RT) followed by quantitative (q)PCR analysis

For reverse transcription (RT) followed by quantitative PCR (qPCR) analysis, 500 ng of total RNA was used. For qPCR analysis, 0.1 μl cDNA was employed with 250 nM of primers (Supplementary File 1) and KAPA SYBR^®^ FAST qPCR Kits (KAPA Biosystems) as described [39]. Divergent primers spanning the circRNA junctions of interest were designed using CircInteractome [40]. RT-qPCR analysis was carried out on a QuantStudio 5 Real-Time PCR System (Thermo Fisher Scientific) with a cycle setup of 3 min at 95°C and 40 cycles of 5 s at 95°C, and 20 s at 60°C. For the mRNAs examined, primers were designed in exons different from those predicted to constitute the circRNA body. Relative RNA levels were calculated after normalizing to the levels of *RPS9* mRNA (human) or *Rps9* mRNA (mouse) using the 2^−ΔΔCt^ method. The levels of housekeeping *TBP* mRNA (human) or *Tbp* mRNA (mouse) were measured as additional controls. The same procedure was followed in both human and mouse samples.

### Bioinformatic analysis of sequencing data

BCL files were de-multiplexed and converted to FASTQ files using bcl2fastq program (v2.20.0.422). FASTQ files were trimmed for adapter sequences using Cutadapt version v1.18 and aligned to human genome hg19 Ensembl v82 using STAR software v2.4.0j [41]; featureCounts (v1.6.4) [42] were used to create gene counts from the samples for linear RNA analysis. The chimeric junction file obtained from STAR software was parsed for fusion junctions and analyzed using CIRCexplorer v1.1.10 [43] to obtain the circularizing junction counts for circRNA analysis as well as for circRNA annotation. The RNA sequencing data are deposited in GSE215424.

For both the linear and the circRNA transcripts, read counts were normalized and differential abundance between healthy and ALS individuals was assessed using the DESeq2 package version 1.30.0 pipeline [44] in R (version 4.0.3). Briefly, the dispersion of samples was explored by using a principal component analysis (PCA) scattergram of regularized logarithm (rlog) transformed counts. Statistical testing was performed using the Wald test. Linear RNAs were defined as differentially regulated with an absolute log2 fold change > 1 and Benjamini–Hochberg adjusted *p*-value < 0.05. Given the low counts and highly variable abundance of circRNAs, we focused on identifying only abundant circRNAs by requiring one or more counts in at least 40% of the ALS muscle samples. Using this stringent cutoff, 250 circRNA transcripts were selected and subjected to further analysis based on their fold change; significance was established based on an unadjusted *p*-value < 0.05.

### Immunostaining

At day 32, iPSCs differentiated in 96-well plates were fixed in 4% (v/v) paraformaldehyde (PFA) and then rinsed with phosphate-buffered saline (PBS). Fixed cultures were blocked in 5% (v/v) donkey serum with 0.03% (v/v) Triton X-100 and 2% fetal bovine serum (FBS). Blocked cell preparations were incubated with primary antibodies anti-SMI32 (BioLegend 801701, dilution 1:250) raised in mouse and anti-Isl1 (Abcam ab109517, dilution 1:500) raised in rabbit. After overnight incubation with the primary antibodies at 4°C, fixed cells were rinsed with PBS and incubated in species-specific secondary antibodies conjugated with Alexa Fluor 488 or Alexa Fluor 594 (dilution 1:1000) at room temperature for 2 h. Nuclei were counterstained with DAPI followed by rinsing with PBS. Cells were visualized using a fluorescent microscope (EVOS M5000) at 10× magnification.

### Statistical analysis

Quantitative data are represented as the means ± SD of the number of samples indicated in each case; statistical significance was established using unpaired Student’s *t*-test in GraphPad Prism (9.0). A *p*-value of < 0.05 was considered statistically significant and was indicated in the figures as ^*^p < 0.05, ^**^p < 0.01, ^***^p < 0.001. Graphs were generated using GraphPad Prism (9.0).

## RESULTS

### Transcriptomic analysis of normal and ALS skeletal muscle

**Figure 1:**
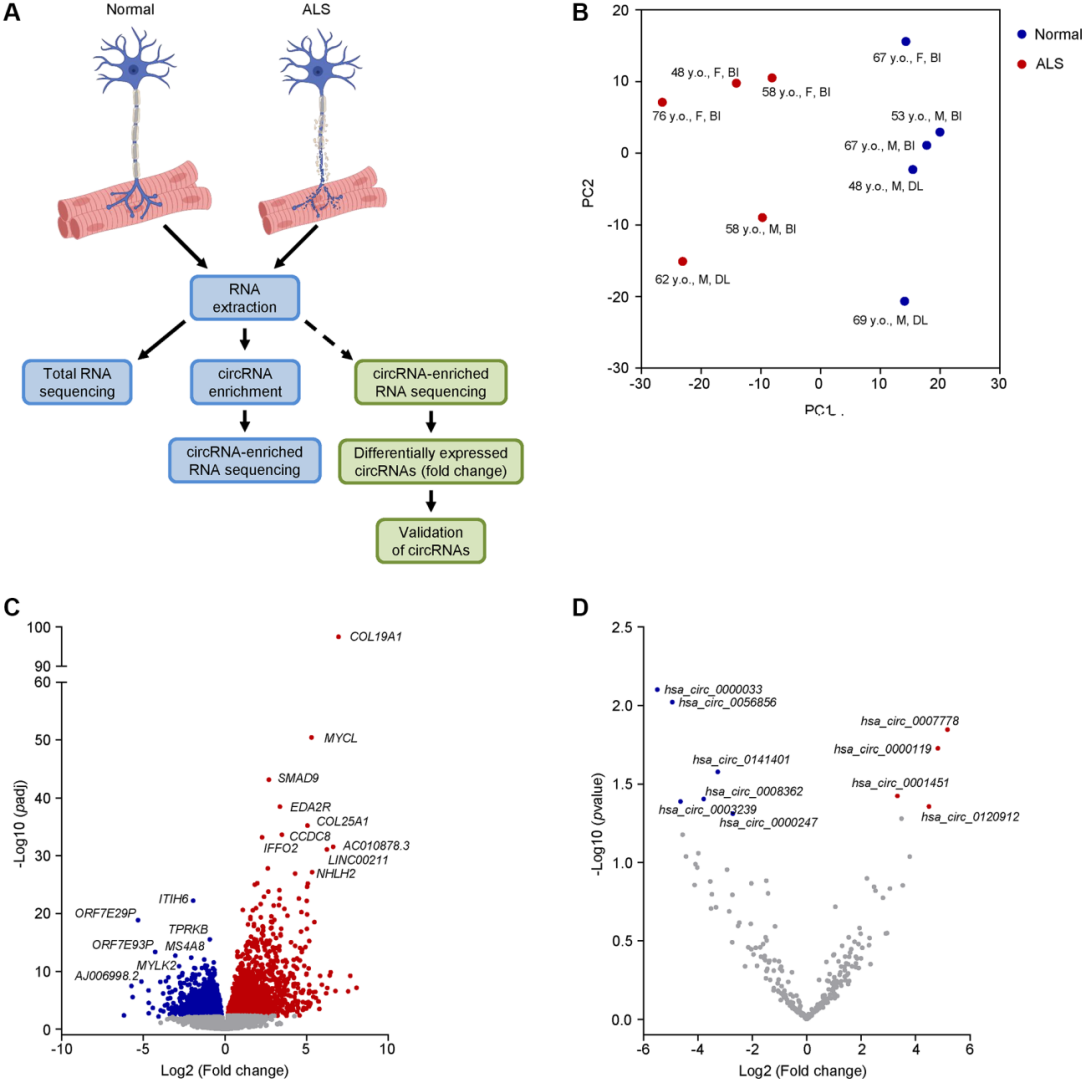
**Transcriptomic profiling and differential expression of linear and circRNA transcripts in ALS muscle.** (**A**) Schematic of the RNA-seq workflow (blue boxes) and validation of candidate circRNAs (green boxes). (**B**) PCA performed on linear coding and long noncoding transcripts of human muscle biopsies from 5 normal and 5 ALS individuals. The age, sex, and muscle biopsy site are also listed (Abbreviations: y.o.: year old; F: female; M: male; BI: biceps brachii; DL: deltoid). (**C**, **D**) Differential expression of linear coding and noncoding transcripts (**C**) or circRNAs (**D**) in the ALS cohort by RNA-seq analysis. For (**C**), significance was established based on adjusted *p*-value < 0.05, whereas for (**D**) significance was established based on an unadjusted *p*-value < 0.05.

To begin to identify differentially expressed RNAs in ALS, we used muscle biopsy samples from ALS patients followed in our clinic with a definite diagnosis of ALS (Table 1). Histologically normal muscle biopsies were used as a control. For later validation of our findings, we also included additional normal and ALS muscle biopsies, as well as muscle samples from neuropathy and myopathy disease controls (Table 1 and Supplementary Table 1). We isolated total RNA from muscle biopsies (5 healthy and 5 ALS individuals) and performed total RNA sequencing (RNA-seq) analysis (Figure 1A). The RNA sequencing data are deposited in GSE215424. Principal component analysis (PCA) using the linear transcripts revealed a distinct separation of the ALS cohort compared to the normal cohort (Figure 1B).

**Table 1:** Demographic and clinical data for muscle biopsies.

	**Normal**	**ALS**	**Myopathy**	**Neuropathy**
**Number**	12	8	8	5
**Mean age (years) ^a^**	59 ± 9	64 ± 11	62 ± 15	50 ± 13
**Age range (years)**	46–75	48–77	38–75	33–63
**Sex (M:F)**	8:4	2:6	1:7	3:2
**Duration ^b^ (m)**		13 ± 3		
**Diagnosis**			Inflammatory (5)^c^	Axonal (1)
			Mitochondrial (2)	CIDP (2)
			Inclusion Body (1)	Plexopathy (1)
				Axonal GBS (1)
**Muscle sampled**	TA (2), BI (4), DL (6)	TA (2), BI (5), DL (1)	TA (1), VL (2), BI (1), DL (4)	TA (3), VL (1), DL (1)

Demographic and clinical data for muscle biopsies derived by normal or ALS individuals, as well as disease controls, myopathy and neuropathy, used for RNA-seq analysis and validation in our study. ^a^Mean age (±SD) at time of sample collection. ^b^Mean duration (±SD) from onset of symptoms to sample collection. ^c^One sample had concomitant histological features of mitochondrial myopathy. Abbreviations: BI: biceps brachii; CIDP: chronic inflammatory demyelinating polyradiculoneuropathy; DL: deltoid; GBS: Guillain Barre syndrome; TA: tibialis anterior; VL: vastus lateralis.

To gain a better insight into the ALS cohort, we assessed the differentially expressed linear coding and noncoding transcripts (Figure 1C and Supplementary File 2). We found that several mRNAs previously associated with ALS were significantly elevated (adjusted *p*-value < 0.05), including *collagen type XIX, alpha 1* (*COL19A1*) mRNA, previously associated with higher mortality and fast progression [45], and *SMAD9* (also known as *SMAD8*) mRNA, encoding a muscle biomarker that is broadly increased in ALS muscle and tracks disease progression [18]. Other transcripts, including *inter-alpha-trypsin inhibitor heavy chain family member 6* (*ITIH6*) mRNA and *myosin light chain kinase* (*MYLK2*) mRNA, which have roles in muscular function, were significantly (adjusted *p*-value < 0.05) downregulated [46, 47]. Taken together, our ALS cohort shared common transcriptomic features with previous reports, supporting its suitability for further assessment of circRNAs.

### Differentially expressed circRNAs in normal and ALS skeletal muscle

**Figure 2:**
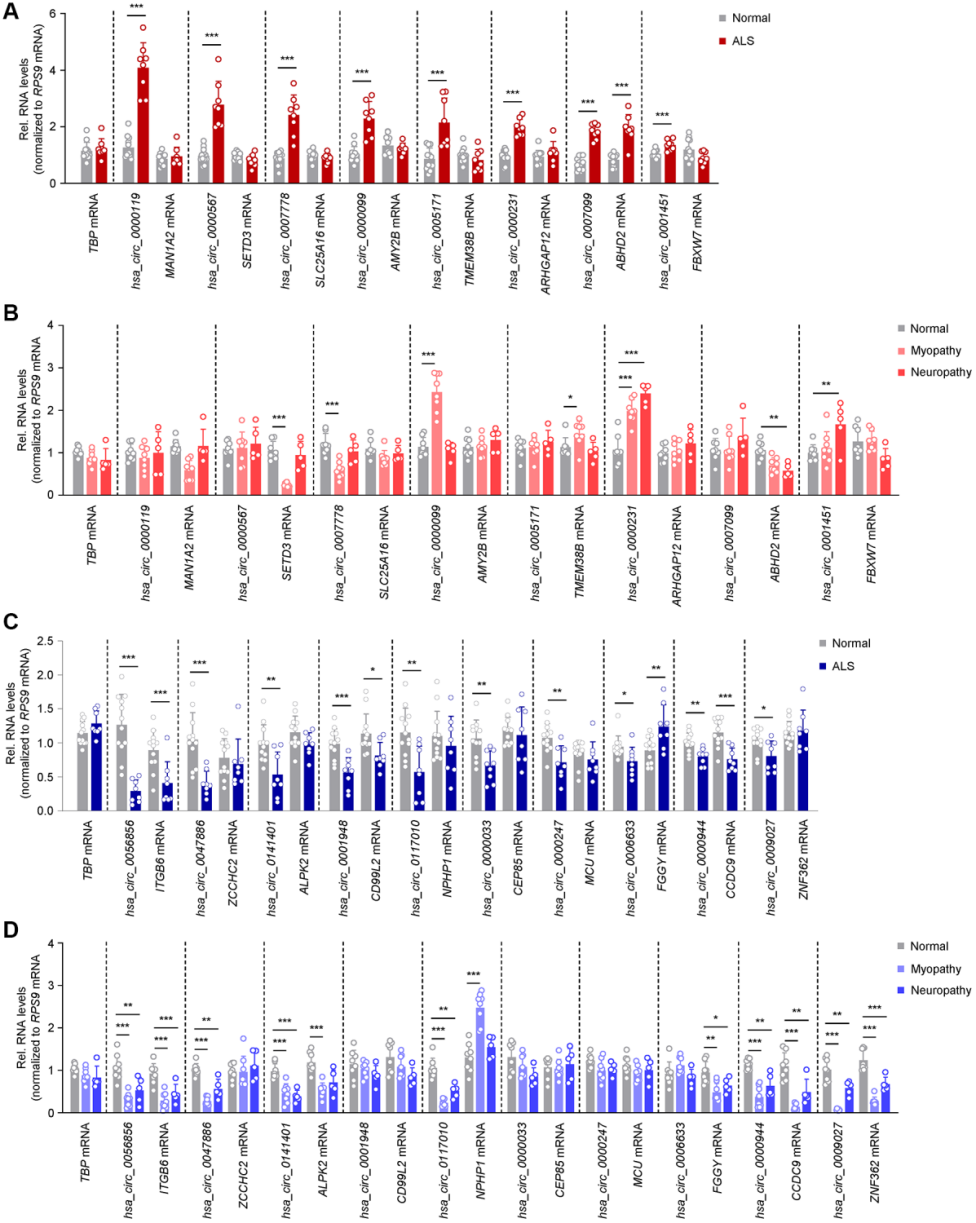
**Differentially abundant circRNAs in ALS skeletal muscle.** (**A**) Levels of significantly elevated circRNAs and their linear counterparts in normal (*n* = 12) and ALS (*n* = 8) muscle biopsies, as assessed by RT-qPCR analysis. (**B**) Levels of expression of the circRNAs and linear counterparts validated in the ALS cohort (**A**), as measured in normal (*n* = 8), myopathy (*n* = 8), and neuropathy (*n* = 5) muscle samples by RT-qPCR analysis. (**C**) Levels of significantly reduced circRNAs and linear counterparts in normal (*n* = 12) and ALS (*n* = 8) muscle biopsies, as assessed by RT-qPCR analysis. (**D**) Levels of expression of the circRNAs and linear counterparts validated in the ALS cohort (**C**), as measured in normal (*n* = 8), myopathy (*n* = 8), and neuropathy (*n* = 5) samples by RT-qPCR analysis. Data were normalized to *RPS9* mRNA levels, whereas *TBP* mRNA levels were included as controls; *p*-values ^*^p < 0.05, ^**^p < 0.01, ^***^p < 0.001.

To identify circRNAs in the muscle biopsies, we enriched our RNA samples by first digesting them with RNase R and then polyadenylating any remaining 3’-OH ends in order to eliminate any residual linear RNAs, as explained ([38] and Materials and Methods). RNA-seq analysis of the circRNA-enriched samples initially identified 4,656 unique junctions; however, after adopting a stringent cutoff criterion requiring at least one junction count in at least 40% of the donors (Material and Methods), we narrowed the results to only 250 circRNAs (Supplementary Figure 1 and Supplementary File 3). Among them, only 4 and 6 circRNAs were significantly (unadjusted *p*-value < 0.05) up- or downregulated, respectively (Figure 1D). Considering the notorious difficulties in detecting circRNAs by RNA-seq analysis and the intrinsic heterogeneity of ALS cohorts, we decided to test the differential expression of at least 50 circRNAs, 25 increased (Supplementary Figure 2A) and 25 decreased (Supplementary Figure 2B), that showed more pronounced fold changes in the RNA-seq analysis using a broader sampling of circRNAs in ALS muscle biopsies. Using the CircInteractome [40] and circBase [48] databases, we identified the circRNA aliases, as well as the counterpart mRNAs arising from the shared linear precursor transcripts, and performed RT-qPCR analysis to assess both forms, the circRNA and the corresponding mRNA. Among the tested circRNAs, 8 were significantly upregulated whereas 10 were significantly downregulated in the ALS cohort (Figure 2); the additionally tested circRNAs did not significantly change or changed in a direction that was opposite to that suggested by the RNA-seq analysis (Supplementary Figure 3). Notably, among the differentially expressed circRNAs based on RT-qPCR analysis, 3 and 4 were predicted to be significantly upregulated or downregulated, respectively, based on the RNA-seq analysis.

For the upregulated group, 7 out of 8 circRNAs, *hsa_circ_0000119*, *hsa_circ_0000567*, *hsa_circ_0007778*, *hsa_circ_0000099*, *hsa_circ_0005171*, *hsa_circ_0000231*, and *hsa_circ_0001451*, were altered without significant changes in the linear counterpart transcripts (*MAN1A2*, *SETD3*, *SLC25A16*, *AMY2B*, *TMEM38B*, *ARHGAP12*, and *FBXW7* mRNAs, respectively) suggesting that these circRNAs may have a role in ALS, independent of the associated mRNAs; only *hsa_circ_0007099* and its counterpart (*ABHD2* mRNA) showed joint increases in abundance (Figure 2A). Interestingly, *hsa_circ_0000567* was previously reported as a blood biomarker in ALS [49]. To evaluate if these circRNAs were specifically elevated in ALS, we assessed their expression levels in muscle biopsies from control myopathy and neuropathy samples (Table 1 and Figure 2B). Notably, the levels of *hsa_circ_0000119*, *hsa_circ_0000567*, *hsa_circ_0007778*, *hsa_circ_0005171*, and *hsa_circ_0007099* did not change significantly (or changed in the opposite direction) in these disease controls, supporting the possibility that their elevation was specific to ALS. Other circRNAs and/or mRNA counterparts changed in myopathy or neuropathy, suggesting that perhaps their altered levels in ALS were linked to common pathways of neuromuscular dysfunction.

For the downregulated circRNAs, 6 out of 10 circRNAs in ALS muscle biopsies, *hsa_circ_0047886*, *hsa_circ_0141401*, *hsa_circ_0117010*, *hsa_circ_0000033*, *hsa_circ_0000247*, and *hsa_circ_0009027*, were selectively reduced while the linear counterparts (*ZCCHC2*, *ALPK2*, *NPHP1*, *CEP85*, *MCU*, and *ZNF362* mRNAs, respectively) were not. In addition, for *hsa_circ_0056856*, *hsa_circ_0001948*, and *hsa_circ_0000944*, both the circRNA and the linear counterparts (*ITGB6*, *CD99L2*, and *CCDC9* mRNAs, respectively) decreased, whereas *hsa_circ_0006633* also decreased while the linear counterpart (*FGGY* mRNA) increased (Figure 2C). Interestingly, many of the downregulated circRNAs were also lower in myopathy and neuropathy disease controls, including *hsa_circ_0056856*, *hsa_circ_0047886*, *hsa_circ_0141401*, *hsa_circ_0117010*, *hsa_circ_0000944*, and *hsa_circ_0009027* (Figure 2D). Taken together, we have identified 7 selectively increased and 6 decreased circRNAs in ALS muscle biopsies, without changes in their counterpart mRNAs; neuropathy and myopathy controls had distinct patterns of expression, sometimes mirroring the differential expression of the circRNAs, sometimes not.

### ALS muscle circRNA patterns are distinct from ALS central nervous system (CNS) circRNA patterns

**Figure 3:**
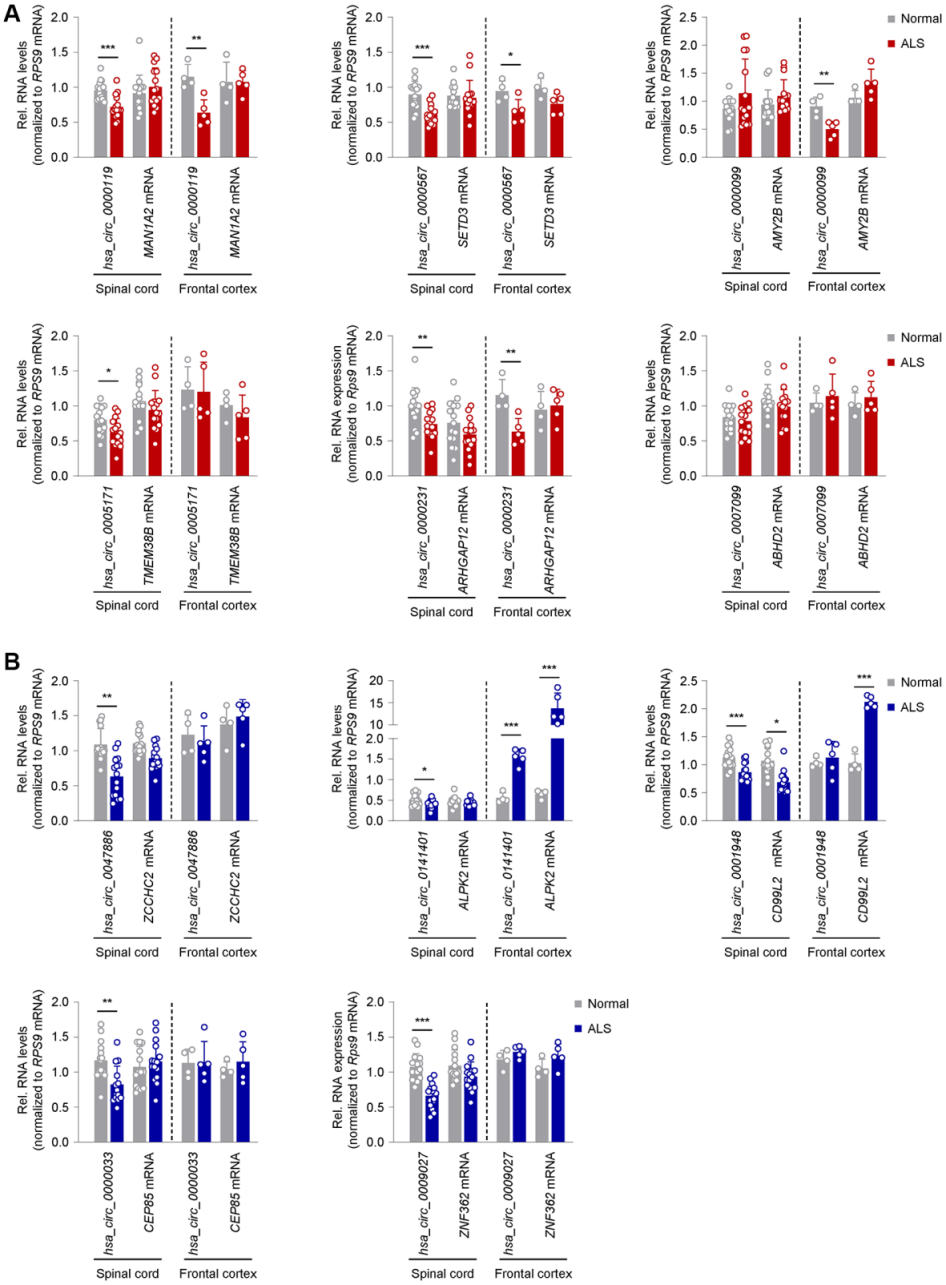
**Expression patterns in ALS CNS of circRNAs previously validated as altered in ALS skeletal muscle.** (**A**) Differential expression of a subset of circRNAs and linear counterparts in human spinal cord (cervical, thoracic, lumbar regions; *n* = 5 for each region in both normal and ALS samples) and frontal cortex (*n* = 4 normal and *n* = 5 ALS), that had been validated as being upregulated in ALS skeletal muscle in Figure 2A. (**B**) Differential expression of a subset of circRNAs and linear counterparts in human spinal cord (cervical, thoracic, lumbar regions; *n* = 5 for each region in both normal and ALS samples) and frontal cortex (*n* = 4 normal and *n* = 5 ALS), that had been validated as being downregulated in ALS skeletal muscle in Figure 2C. Data were normalized to *RPS9* mRNA levels; *p*-values ^*^p < 0.05, ^**^p < 0.01, ^***^p < 0.001.

Given the extensive connectivity between the peripheral neuromuscular system and the CNS, we assessed the levels of the validated circRNAs (Figure 2) in post-mortem spinal cord (SC) and frontal cortex (FC) tissues from normal and ALS individuals (Table 2 and Figure 3). From the group of upregulated muscle circRNAs, none was increased in either SC or FC (Figure 3A and Supplementary Figure 4A). In fact, several circRNAs, including *hsa_circ_0000119*, *hsa_circ_0000567*, *hsa_circ_0005171*, and *hsa_circ_0000231*, showed modest but significant reductions in SC; *hsa_circ_0000119*, *hsa_circ_0000567*, and *hsa_circ_0000231* also decreased in FC. One circRNA, *hsa_circ_0000099*, was unchanged in SC but decreased in FC; none of the counterpart mRNAs in the entire group was altered in SC or FC (Figure 3A and Supplementary Figure 4A).

**Table 2:** Demographic and clinical data for post-mortem spinal cord and cortical specimens.

	**Normal**	**ALS**
**Number**	5	5
**Mean age (years)^a^**	67 ± 14	63 ± 16
**Age range (years)**	52–90	40–81
**Sex (M:F)**	3:2	4:1
**Duration^b^ (m)**		52

Demographic and clinical data for post-mortem spinal cord and cortical specimens derived by normal or ALS individuals used for validation in our study. Specimens were collected from the cervical, thoracic, and lumbar regions, as well as the frontal cortex. ^a^Mean age (±SD) at time of death. ^b^Mean duration (±SD) from onset of symptoms to post-mortem collection.

For the downregulated muscle circRNAs, on the other hand, 8 circRNAs were similarly reduced in SC, including *hsa_circ_0047886*, *hsa_circ_0141401*, *hsa_circ_0001948*, *hsa_circ_0000033*, *hsa_circ_0009027*, *hsa_circ_0006633*, *hsa_circ_0000944*, and *hsa_circ_0009027*, whereas *hsa_circ_0056856* and *hsa_circ_0117010* were unchanged (Figure 3B and Supplementary Figure 4B). Surprisingly, in FC, *hsa_circ_0056856*, *hsa_circ_0141401*, and *hsa_circ_0000944*, as well as their corresponding mRNAs, were significantly increased (Figure 3B and Supplementary Figure 4B). In summary, there was an interesting disparity in the circRNAs elevated in ALS muscle (Figure 2A), as their expression was lower or unchanged in ALS SC and/or FC (Figure 3A); in contrast, circRNAs reduced in ALS muscle (Figure 2C) were generally unchanged or reduced in ALS CNS (Figure 3B).

### Shared circRNA profiles between ALS muscle and ALS iPSC-derived motor neurons

**Figure 4:**
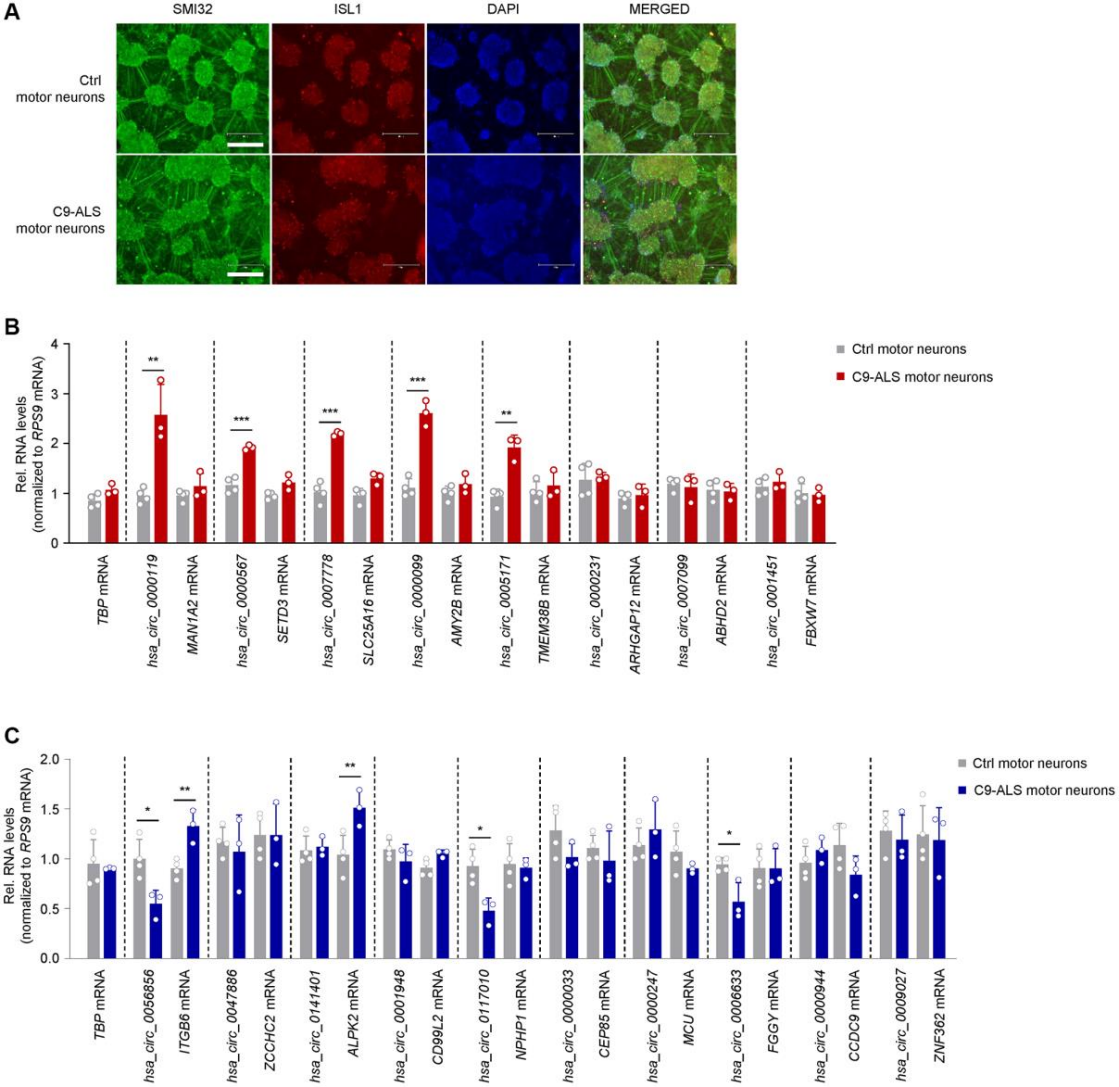
**Expression patterns of ALS muscle circRNAs in iPSC-derived motor neurons bearing ALS-associated *C9ORF72* mutations.** (**A**) Representative images of day-32 motor neurons derived from iPSCs of a healthy subject (CS83iCTR – Table 3) and a C9-ALS patient (CS28iALS – Table 3). Motor neurons were stained for SMI32 and ISL1, nuclei were stained with DAPI. Scale bar indicates, 300 μm. (**B**, **C**) RT-qPCR analysis of iPSC-derived motor neurons from healthy controls (*n* = 4) and ALS patients bearing *C9ORF72* mutations (*n* = 3) to measure the levels of circRNAs that were differentially upregulated (**B**) or downregulated (**C**) in human ALS muscle (Figure 2). Data were normalized to *RPS9* mRNA levels, and *TBP* mRNA levels were included as controls; *p*-values ^*^p < 0.05, ^**^p < 0.01, ^***^p < 0.001.

We extended our analysis to motor neurons derived from iPSCs of ALS patients carrying hexanucleotide repeat expansions in *C9ORF72* (C9-ALS) (Table 3 and Figure 4). iPSC-derived motor neurons from healthy subjects served as control cells. We performed immunocytochemistry on day-32 neurons and found that they expressed two markers of mature motor neurons, ISL1 and SMI32, confirming their motor neuron phenotype (Figure 4A). Among those circRNAs upregulated in ALS skeletal muscle, C9-ALS motor neurons showed similar increases in *hsa_circ_0000119*, *hsa_circ_0000567*, *hsa_circ_0007778*, *hsa_circ_0000099*, and *hsa_circ_0005171* relative to control motor neurons, while the mRNA counterparts for these circRNAs were not altered; the greatest fold change was seen for *hsa_circ_0000119* and *hsa_circ_0000099* (Figure 4B). The other circRNAs that were upregulated in skeletal muscle in the ALS cohort did not change in the ALS motor neurons. Regarding the circRNAs that were reduced in ALS muscle, *hsa_circ_0056856*, *hsa_circ_0117010*, and *hsa_circ_0006633* showed a similar decrease in motor neurons; interestingly, the mRNA counterparts of *hsa_circ_0056856* and *hsa_circ_0141401* (*ITGB3* and *ALPK2* mRNAs, respectively) were modestly but significantly elevated in the ALS motor neurons, in contrast to what was observed in skeletal muscle (Figure 4C). Most other circRNAs reduced in ALS muscle did not significantly decrease in ALS iPSCs (Figure 4C). Taken together, the pattern of increased circRNAs in iPSC-derived motor neurons of ALS patients shared patterns similar to those observed in ALS skeletal muscle.

**Table 3:** Clinical data on ALS patients and control subjects for iPSCs.

**iPSC line**	**Sex**	**Age**	**Clinical**	**Mutation**	**Cell type**
CS0002iCTR	M	51	Normal	N/A	PBMCs
CS83iCTR	F	21	Normal	N/A	Fibroblasts
CS0188iCTR	M	80	Normal	N/A	PBMCs
CS014iCTR	F	52	Normal	N/A	Fibroblasts
CS28iALS	M	47	ALS	C9ORF72 (600–800 repeats)	Fibroblasts
CS29iALS	M	47	ALS	C9ORF72 (600–800 repeats)	Fibroblasts
CS52iALS	M	49	ALS	C9ORF72 (600–800 repeats)	Fibroblasts

Clinical data on ALS patients and control subjects for iPSCs used for validation in our study. Maturation stage of the motor neurons was established by immunocytochemistry on day 32.

### Muscle circRNAs can track disease progression in the SOD1^G93A^ mouse

**Figure 5:**
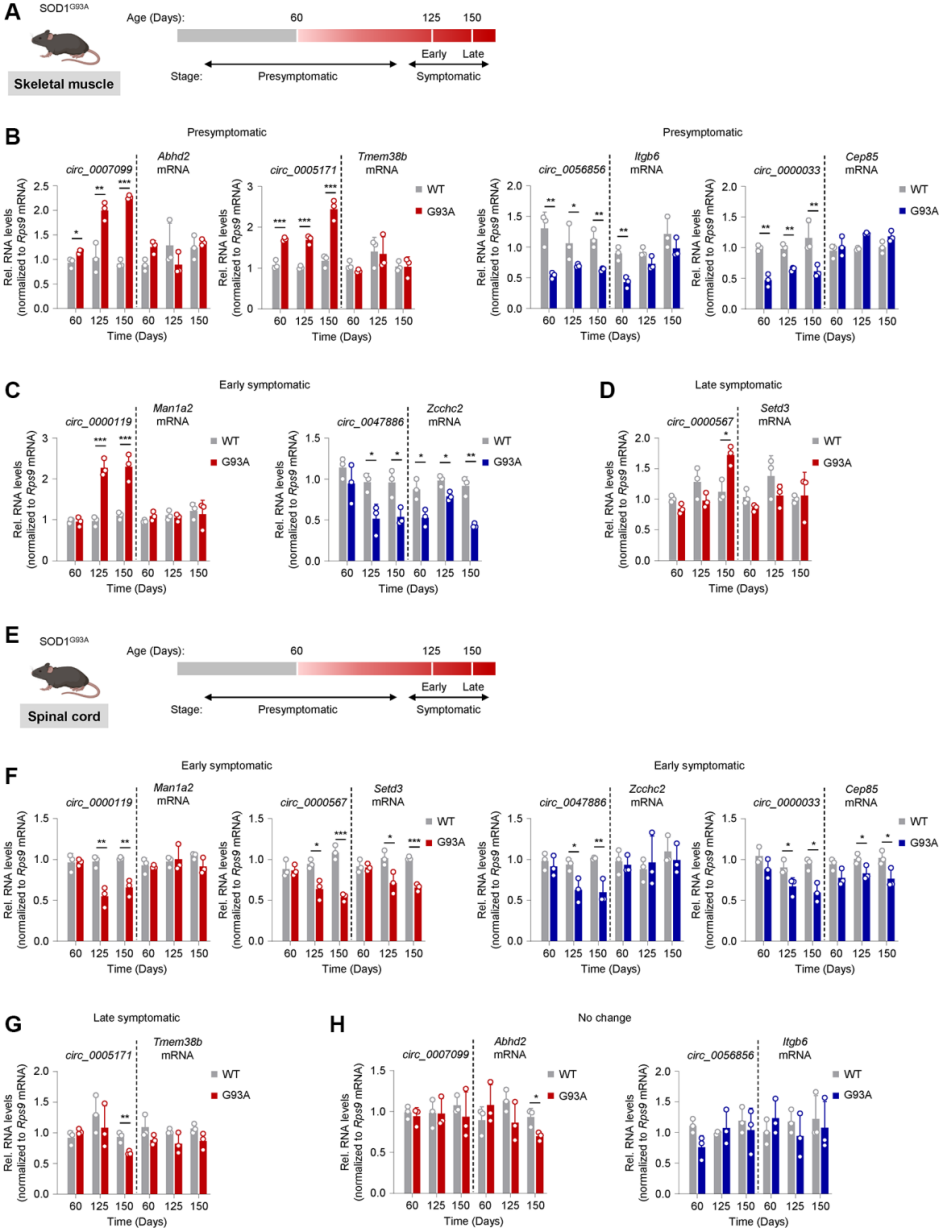
**Tracking circRNA levels in skeletal muscle and spinal cord tissue from the SOD1^G93A^ ALS mouse model.** (**A**) Schematic of disease progression in a genetic ALS mouse model (SOD1^G93A^), from which the gastrocnemius muscle was harvested. Based on rotarod performance and weight measurements, mice are presymptomatic by day 60, early symptomatic by day 125, and late symptomatic by day 150. (**B**–**D**) Shown are the predicted mouse orthologs of circRNAs differentially expressed in human ALS muscle that were found differentially abundant in mouse skeletal muscle at the presymptomatic (**B**), early symptomatic (**C**), and late symptomatic (**D**) stages. (**E**) Schematic of disease progression in a genetic ALS mouse model (SOD1^G93A^), from which spinal cord tissue was harvested. (**F**–**H**) Shown are the predicted mouse orthologs of circRNAs differentially expressed in human ALS muscle that were found differentially abundant in spinal cord at the early (**F**) and late symptomatic (**G**) stage; one circRNA did not change significantly (**H**). The circRNAs are represented in red (upregulated) or blue (downregulated) following their patterns of abundance in human ALS muscle biopsies (Figure 2). Data were normalized to *Rps9* mRNA levels; *p*-values ^*^p < 0.05, ^**^p < 0.01, ^***^p < 0.001.

The SOD1^G93A^ transgenic mouse recapitulates many features of ALS including progressive weakness, motor neuron loss, and muscle denervation compared to age-matched wild-type (WT) littermate controls, thus providing an opportunity to assess temporal patterns of biomarkers with disease progression [50–52]. We assessed the expression of altered circRNA candidates in mouse skeletal muscle at three ages which reflect different disease stages based on rotarod performance and weight measurements [18]: 60 days old [presymptomatic (PS)], 125 days old [early symptomatic (ES)], and 150 days old [late symptomatic (LS)] (Figure 5A, *skeletal muscle*). We first identified mouse circRNA orthologs using the flanking exons of the human circRNA candidates to find the conserved exons in the respective mouse linear ortholog. We then designed divergent primers spanning the predicted junction in mice. A summary of the aliases of the host genes and exons predicted to constitute the circRNA bodies in each species, as well as the predicted sequence overlaps are shown in Supplementary Figure 5.

At the PS stage, four mouse circRNA orthologs, *circ_0007099*, *circ_0005171*, *circ_0056856*, and *circ_0000033*, showed differential expression in gastrocnemius muscle compared to age-matched WT controls (Figure 5B). *Circ_0007099* and *circ_0005171* were upregulated in the SOD1^G93A^ mouse, similar to the human circRNA ortholog, and gradually increased with disease progression through the LS stage, whereas the respective mRNA counterparts were unchanged. Likewise, *circ_0056856* and *circ_0000033* were downregulated in the SOD1^G93A^ mouse, again similar to what was seen for the human orthologs, but the suppression was relatively constant through the LS stage. With the exception of the PS stage for *circ_0056856*, the corresponding mRNAs were unchanged. In the ES stage, *circ_0000119* and *circ_0047886* started to show differential expression in the same direction as in human ALS muscle, with the former showing upregulation and the latter showing downregulation; notably, the expression changes did not progress in the LS stage (Figure 5C). Similar to the human ortholog, the linear counterpart of *circ_0000119* (*Man1a2* mRNA) was unchanged compared to WT, whereas the linear counterpart of *circ_0047886* (*Zcchc2* mRNA) was significantly lower (Figure 5C). *Circ_0000567*, previously reported as a biomarker in ALS [49], was the only one differentially expressed at the LS stage without a change in the mRNA counterpart (Figure 5D); the circRNA levels increased in ways similar to those observed in human ALS muscle. The other candidates, predicted to either increase or decrease in human ALS muscle, showed no differential expression at any stage (Supplementary Figure 6).

We then assessed whether the spinal cord tissues reflected the differential expression of the mouse muscle circRNAs that showed disease stage-associated patterns (Figure 5E, *spinal cord*). Four circRNAs, *circ_0000119*, *circ_0000567*, *circ_00047886*, and *circ_0000033*, showed differential expression at the ES stage. Two of the upregulated muscle circRNAs, *circ_0000119* and *circ_0000567*, were lower in the spinal cord, similar to their human counterparts at the ES stage; the reduced levels persisted but did not further decline through the LS stage. The levels of the linear counterpart of *circ_0000119* (*Man1a2* mRNA) did not change, while the levels of the linear counterpart of *circ_0000567* (*Setd3* mRNA) decreased in parallel through the LS stage (Figure 5F). The downregulated muscle circRNAs *circ_0047886* and *circ_0000033* decreased in the spinal cord similar to what was seen for the human orthologs. The linear counterpart for *circ_0000033* (*Cep85* mRNA) also decreased, albeit modestly, in contrast to what we saw in human ALS spinal cord (Figure 5F). Lastly, *circ_0005171* was downregulated in the LS stage (Figure 5G), whereas *circ_0007099* and *circ_0056856* did not change at any disease stage (Figure 5H). Among other prominent muscle circRNA candidates, *circ_0141041* showed differential expression at the PS stage and progressively decreased through the LS stage (Supplementary Figure 7A), whereas *circ_0000231*, *circ_0000099*, *circ_0001948*, and *circ_0000944* decreased at the LS stage in spinal cord (Supplementary Figure 7B). Other candidate muscle circRNAs had variable patterns or no significant change (Supplementary Figure 7C).

A summary of the changes for circRNAs based on RT-qPCR analysis in human and mouse tissues is shown in Table 4. Several circRNAs showed consistent changes in human and mouse skeletal muscle (5 upregulated and 4 downregulated circRNAs). Several circRNAs also showed consistent changes (all decreased) in human and mouse spinal cord tissues, including *hsa_circ_0000119*, *hsa_circ_0000567*, *hsa_circ_0005171*, *hsa_circ_0047886*, *hsa_circ_0001948*, and *hsa_circ_0000033*. We note an interesting difference in direction of abundance, with those circRNAs elevated in muscle (top half of Table 4) showing reduced abundance in spinal cord.

**Table 4:** Summary of circRNA patterns in ALS patients and the SOD1^G93A^ mouse^a^.

**circRNA**	**Muscle**			**Spinal cord**		**Cortex**	**iPSCs**
	**ALS**	**^b^Non-ALS**	**^c^SOD1^G39A^**	**ALS**	**SOD1^G39A^**	**ALS**	**C9ORF72**
*hsa_circ_0000119*	*↑*	*–*	*↑ (ES)*	*↓*	*↓ (ES)*	*↓*	*↑*
*hsa_circ_0000567*	*↑*	*–*	*↑ (LS)*	*↓*	*↓ (ES)*	*↓*	*↑*
*hsa_circ_0007778*	*↑*	*↓ (M)*	*–*	*–*	*–*	*–*	*↑*
*hsa_circ_0000099*	*↑*	*↑ (M)*	*–*	*–*	*↓ (LS)*	*↓*	*↑*
*hsa_circ_0005171*	*↑*	*–*	*↑ (PS)*	*↓*	*↓ (LS)*	*–*	*↑*
*hsa_circ_0000231*	*↑*	*↑ (M, N)*	*–*	*↓*	*↓ (LS)*	*↓*	*–*
*hsa_circ_0007099*	*↑*	*–*	*↑ (PS)*	*–*	*–*	*–*	*–*
*hsa_circ_0001451*	*↑*	*↑ (N)*	*–*	*–*	*–*	*–*	*–*
***hsa_circ_0056856***	***↓***	***↓ (M, N)***	***↓ (PS)***	***–***	***–***	***↑***	***↓***
***hsa_circ_0047886***	***↓***	***↓ (M, N)***	***↓ (ES)***	***↓***	***↓ (ES)***	***–***	***–***
***hsa_circ_0141401***	***↓***	***↓ (M, N)***	***↓ (ES)***	***↓***	***↓ (PS)***	***↑***	***–***
***hsa_circ_0001948***	***↓***	***–***	***–***	***↓***	***↓ (LS)***	***–***	***–***
***hsa_circ_0117010***	***↓***	***↓ (M, N)***	***–***	***–***	***–***	***–***	***↓***
***hsa_circ_0000033***	***↓***	***–***	***↓ (PS)***	***↓***	***↓ (ES)***	***–***	***–***
***hsa_circ_0000247***	***↓***	***–***	***↑ (LS)***	***↓***	***–***	***–***	***–***
***hsa_circ_0006633***	***↓***	***–***	***–***	***↓***	***–***	***–***	***↓***
***hsa_circ_0000944***	***↓***	***↓ (M, N)***	***–***	***↓***	***↓ (LS)***	***↑***	***–***
***hsa_circ_0009027***	***↓***	***↓ (M, N)***	***–***	***↓***	***↓ (LS)***	***–***	***–***

Summary of differentially up- (Italic) or downregulated (Bold italic) circRNAs in human ALS skeletal muscle and their expression patterns across muscle and spinal cord specimens in human and SOD1^G39A^ mice as well as in human iPSC-derived motor neurons harboring repeat expansions in *C9ORF72*. ^a^All comparisons are to normal biopsies (human) or age-matched WT mouse tissue samples. –: no significant difference. ^b^Non-ALS, human biopsy samples histologically diagnosed as consistent with myopathy (M) or neuropathy (N). ^c^Disease stages in ALS mouse model. Abbreviations: PS: presymptomatic; ES: early symptomatic; LS: late symptomatic.

## DISCUSSION

In this work, we have identified distinct patterns of circRNA expression in human ALS muscle tissue, many appearing to be disease-specific, that display expression gradients at different levels within the CNS. We identified considerable overlap of these patterns in skeletal muscle and CNS tissues of the SOD1^G93A^ mouse, allowing insight into their potential association with different disease stages and progression. The long-term significance of the work includes: (1) the possibility that the dysregulated circRNAs in disease pathology reflect adaptive or maladaptive responses to the degenerative process, and as such, may represent targets for therapeutic development; (2) the possibility that the gradients observed within the CNS and periphery may reflect disease activity such as movement of circRNAs within the motor neurons; and (3) the potential translational application of circRNAs as biomarkers of ALS disease onset and progression.

The peripheral neuromuscular system is active in ALS disease progression, beginning at early presymptomatic stages, and may be the first manifestation of disease pathology [9, 53, 54]. These observations are primarily based on temporal patterns in the SOD1^G93A^ mouse, a model that recapitulates many but not all pathological features of classic ALS [55]. In our prior work, molecular markers identified in human ALS muscle samples by RNA-seq analysis [18] have remarkable overlap with skeletal muscle from the SOD1^G93A^ mouse, suggesting that molecular changes in the peripheral neuromuscular system in ALS represent a common pathway after the degenerative process has been triggered [16–21]. Our findings in this report support this possibility, as there was considerable overlap in the pattern of altered circRNAs in human and mouse ALS muscle and spinal cord tissues (Table 4). The onset of these patterns varied with individual circRNAs over the lifespan of the ALS mouse, suggesting disease stage-specific triggers for their expression. Several circRNAs, including *circ_0007099*, *circ_0005171, circ_0056856,* and *circ_0000033*, were altered at 60 days post-natal, which long precedes rotarod impairment and weight loss [18, 56]. Although molecular and mitochondrial changes in skeletal muscle occur as early as 40 days post-natal [56, 57], the early appearance of these circRNAs indicates that they are part of a molecular program initiated close to the onset of disease. Two of these upregulated circRNAs, *circ_0007099* and *circ_0005171*, increased with age, suggesting the possibility that they are muscle markers of disease progression as we have previously observed with members of the SMAD family, CYP27B1, FRZB, FGF23, and TGF-β1, 2, and 3 [16, 18–21]. One circRNA, *circ_0000119*, increased in the ES stage but remained stably increased in the LS stage.

The utility of these muscle circRNAs as clinical biomarkers would depend on their detectability in blood as serial muscle biopsies are impractical in the clinic in addition to the marked variability of muscles involved in individual ALS patients (unlike in the SOD1^G93A^ mouse). We attempted to quantify the 18 circRNAs in plasma of ALS patients, but the levels were too low for detection (not shown). This limitation may reflect the relative low abundance of circRNAs (compared to microRNAs for example) or methodological limitations in measuring circRNAs in the blood. The other possibility is that the circRNAs remain intracellular or are trapped in the neuromuscular space, as we postulated previously for other ALS muscle biomarkers [16, 19].

**Figure 6:**
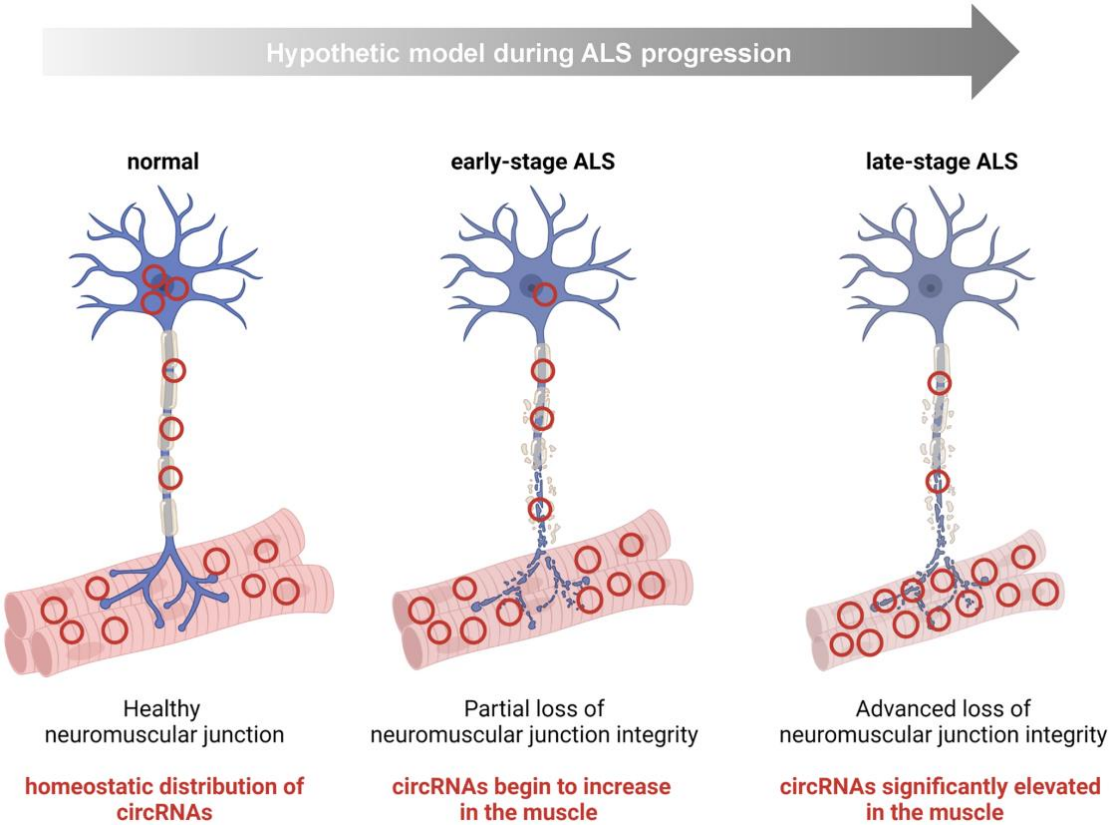
**Hypothesis: some circRNAs mobilize within the motor neuron to the NMJ/muscle during the progression of ALS.** For a handful of human circRNAs identified in this study, namely *hsa_circ_0000119*, *hsa_circ_0000567*, *hsa_circ_0007778*, *hsa_circ_0000099*, and *hsa_circ_0005171*, we documented an increase in ALS muscle and a concomitant decline in ALS CNS tissue. One possibility is that this shift represents a disease-associated mobilization of some circRNAs within motor neurons to the peripheral neuromuscular system (e.g., terminal motor neuron, NMJ, or endomysial space).

Intriguingly, many of the increased muscle circRNAs, *hsa_circ_0000567*, *hsa_circ_0007778*, *hsa_circ_0000099*, and *hsa_circ_0005171*, were also increased in iPSC-derived ALS motor neurons, raising the possibility that the increase in muscle was due to their presence in the terminal motor neuron as part of the disease process (Figure 4 and Figure 6). In support of this possibility, these circRNAs were decreased in ALS spinal cord tissues, whereas the linear counterparts were ubiquitously expressed and unchanged. Although the differential abundance could also be explained in other ways, the preliminary hypothesis of circRNA mobilization warrants further investigation, as it could be investigated if these circRNAs are found in synaptosomes or exosomes present in the neuromuscular junction space. Of interest, a prior study that extensively examined circRNAs in the CNS found that circRNAs were enriched at synapses [58]. In contrast, the consistent pattern of most downregulated circRNAs between muscle, spinal cord, and iPSCs (summarized in Table 4) may reflect a more global effect of the degenerative process.

The functional significance of these differentially abundant circRNAs also remains to be elucidated. Among the vast class of different circRNAs expressed across tissues at various times (which likely exist in the range of tens to hundreds of thousands), a few highly abundant circRNAs have been shown to serve as ‘sponges’ for microRNAs, and in this manner they can derepress mRNAs that are otherwise silenced by these microRNAs (e.g., *CDR1as* and miR-7 [25]). Other circRNAs can bind proteins and function in different transcriptional and post-transcriptional roles (e.g., *circSamd4* and transcription factors PURA/PURB or *circPCNX* and RNA-binding protein AUF1 [34, 39]), and yet other circRNAs have open reading frames with the potential ability to encode functional peptides (e.g., *Circ-ZNF609* [31]). Notably, recent studies have highlighted the importance of RNA-binding protein FUS in ALS [59] and how unique circRNAs, like *Circ-Hdgfrp3*, can shuttle along neurites, which upon stress become trapped in cytoplasmic aggregates in motor neurons carrying mutant FUS [60].

For the 18 circRNAs (8 elevated, 10 reduced in ALS muscle) identified by RT-qPCR analysis in this report, their cellular functions are unknown. At present, circRNA functions cannot be predicted by simply analyzing the circRNA sequences; instead, they must be empirically determined using molecular biology methods that include affinity purification of bound molecules, overexpression and silencing, mutation, and other dedicated approaches. As our studies advance, we will investigate the function of the most promising and abundant circRNAs, among the 18 circRNAs reported here. We are especially interested in those that appeared to be specific for ALS (Figure 2), as they may help to characterize disease-associated molecular pathways that could be targeted therapeutically.

## Supplementary Materials

Supplementary File 1

Supplementary File 2

Supplementary File 3

Supplementary Figures

Supplementary Table 1
