# Supplementary material for: Transcriptomic analysis of human ALS skeletal muscle reveals a disease-specific pattern of dysregulated circRNAs
Source: Aging (Albany NY). 2022 Dec 30;14(24):9832–59. doi: 10.18632/aging.204450 (PMC9831722; doi:10.18632/aging.204450)
Supplement: Supplementary Figures [file aging-14-204450-s004.pdf]

# SUPPLEMENTARY FIGURES

**A**

| circRNA | Log2 fold change | circRNA | Log2 fold change | circRNA | Log2 fold change | circRNA | Log2 fold change | circRNA | Log2 fold change | circRNA | Log2 fold change | circRNA | Log2 fold change |
|---|---|---|---|---|---|---|---|---|---|---|---|---|---|
| hsa_circ_0007778 | 5.179 | hsa_circ_0001369 | 1.983 | hsa_circ_0001900 | 1.455 | hsa_circ_0096442 | 1.087 | hsa_circ_0008732 | 0.827 | hsa_circ_0134651 | 0.559 | hsa_circ_0003253 | 0.193 |
| hsa_circ_0000119 | 4.824 | hsa_circ_0000099 | 1.943 | hsa_circ_0084615 | 1.427 | hsa_circ_0001246 | 1.063 | hsa_circ_0001788 | 0.810 | hsa_circ_0006717 | 0.555 | hsa_circ_0001727 | 0.188 |
| hsa_circ_0120912 | 4.497 | chr2:233587263-233589401 | 1.912 | hsa_circ_0000835 | 1.414 | hsa_circ_0024605 | 1.050 | hsa_circ_0000437 | 0.805 | hsa_circ_0008648 | 0.554 | hsa_circ_0000024 | 0.180 |
| hsa_circ_0000417 | 3.788 | hsa_circ_0004805 | 1.904 | hsa_circ_0004471 | 1.403 | chr4:169432573-169433563 | 1.035 | hsa_circ_0001982 | 0.774 | hsa_circ_0000880 | 0.544 | hsa_circ_0006434 | 0.179 |
| hsa_circ_0005552 | 3.532 | hsa_circ_0004912 | 1.852 | hsa_circ_0007385 | 1.398 | hsa_circ_0008319 | 1.032 | chr12:112757028-112757546 | 0.763 | hsa_circ_0000745 | 0.530 | hsa_circ_0008114 | 0.158 |
| hsa_circ_0000567 | 3.485 | chr12:57637693-57637870 | 1.812 | hsa_circ_0000896 | 1.389 | hsa_circ_0105987 | 1.015 | hsa_circ_0001772 | 0.759 | hsa_circ_0007444 | 0.522 | chr6:54001512-54067031 | 0.140 |
| hsa_circ_0001451 | 3.334 | hsa_circ_0004870 | 1.808 | hsa_circ_0002484 | 1.301 | hsa_circ_0004276 | 1.011 | hsa_circ_0007364 | 0.731 | hsa_circ_0000591 | 0.483 | hsa_circ_0080420 | 0.138 |
| hsa_circ_0002566 | 3.058 | hsa_circ_0005806 | 1.807 | hsa_circ_0001236 | 1.278 | hsa_circ_0004276 | 1.009 | hsa_circ_0117628 | 0.726 | hsa_circ_0003810 | 0.461 | hsa_circ_0002457 | 0.136 |
| hsa_circ_0005615 | 2.955 | hsa_circ_0002538 | 1.762 | hsa_circ_0001020 | 1.271 | chr3:114069120-114070725 | 1.004 | hsa_circ_0004058 | 0.723 | chr16:85667519-85667738 | 0.455 | hsa_circ_0000378 | 0.132 |
| hsa_circ_0002972 | 2.908 | hsa_circ_0007099 | 1.669 | hsa_circ_0000711 | 1.271 | hsa_circ_0006114 | 1.001 | hsa_circ_0000754 | 0.661 | hsa_circ_0001178 | 0.401 | chr3:134327490-134346660 | 0.110 |
| chr4:114376881-114421667 | 2.802 | hsa_circ_0002245 | 1.635 | hsa_circ_0007883 | 1.235 | hsa_circ_0003624 | 0.999 | hsa_circ_0001359 | 0.647 | hsa_circ_0004212 | 0.398 | hsa_circ_0001159 | 0.096 |
| hsa_circ_0008812 | 2.525 | chr6:123696749-123714822 | 1.597 | hsa_circ_0001053 | 1.213 | hsa_circ_0001017 | 0.979 | hsa_circ_0001073 | 0.624 | chr2:152403942-152410539 | 0.397 | hsa_circ_0001173 | 0.076 |
| hsa_circ_0001016 | 2.474 | hsa_circ_0056018 | 1.566 | chr15:63988322-64008672 | 1.202 | hsa_circ_0001445 | 0.979 | hsa_circ_0103069 | 0.623 | hsa_circ_0006916 | 0.363 | hsa_circ_0008832 | 0.076 |
| hsa_circ_0004113 | 2.345 | hsa_circ_0001439 | 1.562 | hsa_circ_0005328 | 1.170 | hsa_circ_0006665 | 0.975 | hsa_circ_0103891 | 0.611 | hsa_circ_0084606 | 0.360 | hsa_circ_0001801 | 0.071 |
| hsa_circ_0000231 | 2.311 | hsa_circ_0000605 | 1.545 | hsa_circ_0006629 | 1.126 | hsa_circ_0000906 | 0.939 | hsa_circ_0003922 | 0.602 | hsa_circ_0001367 | 0.352 | hsa_circ_0070039 | 0.054 |
| hsa_circ_0073517 | 2.289 | hsa_circ_0003270 | 1.524 | hsa_circ_0000296 | 1.121 | hsa_circ_0001329 | 0.923 | hsa_circ_0011536 | 0.598 | hsa_circ_0008103 | 0.343 | hsa_circ_0001684 | 0.026 |
| hsa_circ_0001423 | 2.214 | hsa_circ_0008501 | 1.523 | hsa_circ_0006501 | 1.098 | chr10:126370175-126370948 | 0.887 | hsa_circ_0000643 | 0.596 | hsa_circ_0000384 | 0.275 | hsa_circ_0018493 | 0.020 |
| hsa_circ_0005465 | 2.041 | hsa_circ_0001368 | 1.522 | chr3:37132957-37138151 | 1.095 | hsa_circ_0056280 | 0.853 | hsa_circ_0132246 | 0.583 | hsa_circ_0008494 | 0.197 | hsa_circ_0000586 | 0.014 |
| hsa_circ_0005171 | 1.994 | hsa_circ_0002158 | 1.477 | hsa_circ_0000441 | 1.093 | hsa_circ_0009043 | 0.845 | hsa_circ_0002468 | 0.572 | | | | |

**B**

| circRNA | Log2 fold change | circRNA | Log2 fold change | circRNA | Log2 fold change | circRNA | Log2 fold change | circRNA | Log2 fold change | circRNA | Log2 fold change | circRNA | Log2 fold change |
|---|---|---|---|---|---|---|---|---|---|---|---|---|---|
| hsa_circ_0000033 | -5.490 | hsa_circ_0007646 | -2.740 | hsa_circ_0000002 | -1.542 | hsa_circ_0008604 | -1.067 | hsa_circ_0000734 | -0.778 | hsa_circ_0001092 | -0.5035 | hsa_circ_0000075 | -0.196 |
| hsa_circ_0056856 | -4.939 | hsa_circ_0002590 | -2.734 | chr7:152007050-152012423 | -1.523 | hsa_circ_0000524 | -1.051 | hsa_circ_0007552 | -0.765 | hsa_circ_0006404 | -0.44229 | chr10:69902696-69918384 | -0.189 |
| hsa_circ_0003239 | -4.637 | hsa_circ_0000247 | -2.718 | hsa_circ_0004524 | -1.515 | hsa_circ_0000551 | -1.035 | hsa_circ_0016866 | -0.727 | chr13:42439871-42461497 | -0.42862 | hsa_circ_0005946 | -0.185 |
| hsa_circ_0003274 | -4.559 | hsa_circ_0001953 | -2.524 | chr13:42385360-42393522 | -1.502 | hsa_circ_0008521 | -1.034 | hsa_circ_0001333 | -0.706 | hsa_circ_0002163 | -0.38156 | hsa_circ_0072547 | -0.175 |
| hsa_circ_0116009 | -4.433 | hsa_circ_0006354 | -2.476 | hsa_circ_0001792 | -1.483 | hsa_circ_0001074 | -1.018 | hsa_circ_0000246 | -0.677 | hsa_circ_0000471 | -0.37706 | hsa_circ_0007695 | -0.168 |
| hsa_circ_0006117 | -4.123 | hsa_circ_0001948 | -2.269 | hsa_circ_0003441 | -1.480 | hsa_circ_0002387 | -0.975 | hsa_circ_0008297 | -0.615 | hsa_circ_0001030 | -0.32747 | hsa_circ_0003218 | -0.162 |
| hsa_circ_0047886 | -4.088 | hsa_circ_0040823 | -2.149 | hsa_circ_0078784 | -1.473 | hsa_circ_0003865 | -0.957 | chr3:157839891-157841780 | -0.611 | chr5:137219072-137219280 | -0.29999 | hsa_circ_0000569 | -0.158 |
| hsa_circ_0001148 | -4.026 | hsa_circ_0009027 | -2.055 | chr11:64525250-64526176 | -1.448 | hsa_circ_0001851 | -0.941 | hsa_circ_0001819 | -0.594 | hsa_circ_0008285 | -0.29369 | chr11:22856452-22859910 | -0.155 |
| hsa_circ_0131936 | -3.981 | hsa_circ_0000914 | -2.039 | hsa_circ_0000061 | -1.422 | hsa_circ_0003261 | -0.928 | hsa_circ_0009061 | -0.585 | hsa_circ_0002301 | -0.29046 | hsa_circ_0086414 | -0.126 |
| hsa_circ_0008362 | -3.790 | hsa_circ_0006633 | -2.035 | hsa_circ_0002490 | -1.395 | hsa_circ_0002398 | -0.923 | hsa_circ_0001654 | -0.582 | hsa_circ_0001360 | -0.28341 | hsa_circ_0004502 | -0.118 |
| hsa_circ_0125943 | -3.545 | chr11:1862138-1862238 | -1.960 | chr7:65592690-65599361 | -1.366 | hsa_circ_0001400 | -0.907 | hsa_circ_0117627 | -0.542 | hsa_circ_0063050 | -0.27398 | hsa_circ_0006156 | -0.095 |
| hsa_circ_0067323 | -3.516 | hsa_circ_0018168 | -1.872 | hsa_circ_0007904 | -1.344 | hsa_circ_0007367 | -0.850 | hsa_circ_0008193 | -0.542 | hsa_circ_0135761 | -0.26594 | hsa_circ_0002100 | -0.061 |
| hsa_circ_0067871 | -3.487 | hsa_circ_0001756 | -1.828 | hsa_circ_0002058 | -1.218 | hsa_circ_0001771 | -0.847 | hsa_circ_0003713 | -0.539 | hsa_circ_0105377 | -0.26561 | hsa_circ_0005087 | -0.052 |
| hsa_circ_0000109 | -3.336 | hsa_circ_0000944 | -1.709 | hsa_circ_0128535 | -1.211 | hsa_circ_0001776 | -0.846 | hsa_circ_0108763 | -0.526 | hsa_circ_0001380 | -0.25838 | hsa_circ_0050851 | -0.049 |
| hsa_circ_0141401 | -3.269 | hsa_circ_0000076 | -1.680 | chr6:123637601-123703292 | -1.210 | hsa_circ_0001498 | -0.843 | hsa_circ_0004846 | -0.517 | hsa_circ_0083220 | -0.24715 | chr5:149610867-149624764 | -0.041 |
| hsa_circ_0118236 | -2.926 | hsa_circ_0056019 | -1.606 | hsa_circ_0001136 | -1.186 | hsa_circ_0000043 | -0.827 | hsa_circ_0005993 | -0.511 | hsa_circ_0006107 | -0.23091 | hsa_circ_0000284 | -0.018 |
| hsa_circ_0117010 | -2.849 | hsa_circ_0008086 | -1.568 | hsa_circ_0005600 | -1.161 | hsa_circ_0001861 | -0.814 | hsa_circ_0008368 | -0.509 | hsa_circ_0004823 | -0.20443 | hsa_circ_0008602 | -0.013 |

**Supplementary Figure 1. List of 250 circRNAs expressed in at least 40% of the human normal and ALS skeletal muscle biopsy samples.** Upregulated (**A**) and downregulated (**B**) circRNAs in ALS based on log2 fold change, predicted from our circRNA-enriched RNA-seq analysis of normal (*n* = 5) and ALS (*n* = 5) biopsies. CircRNA nomenclature is based on the CircInteractome or circBase databases.

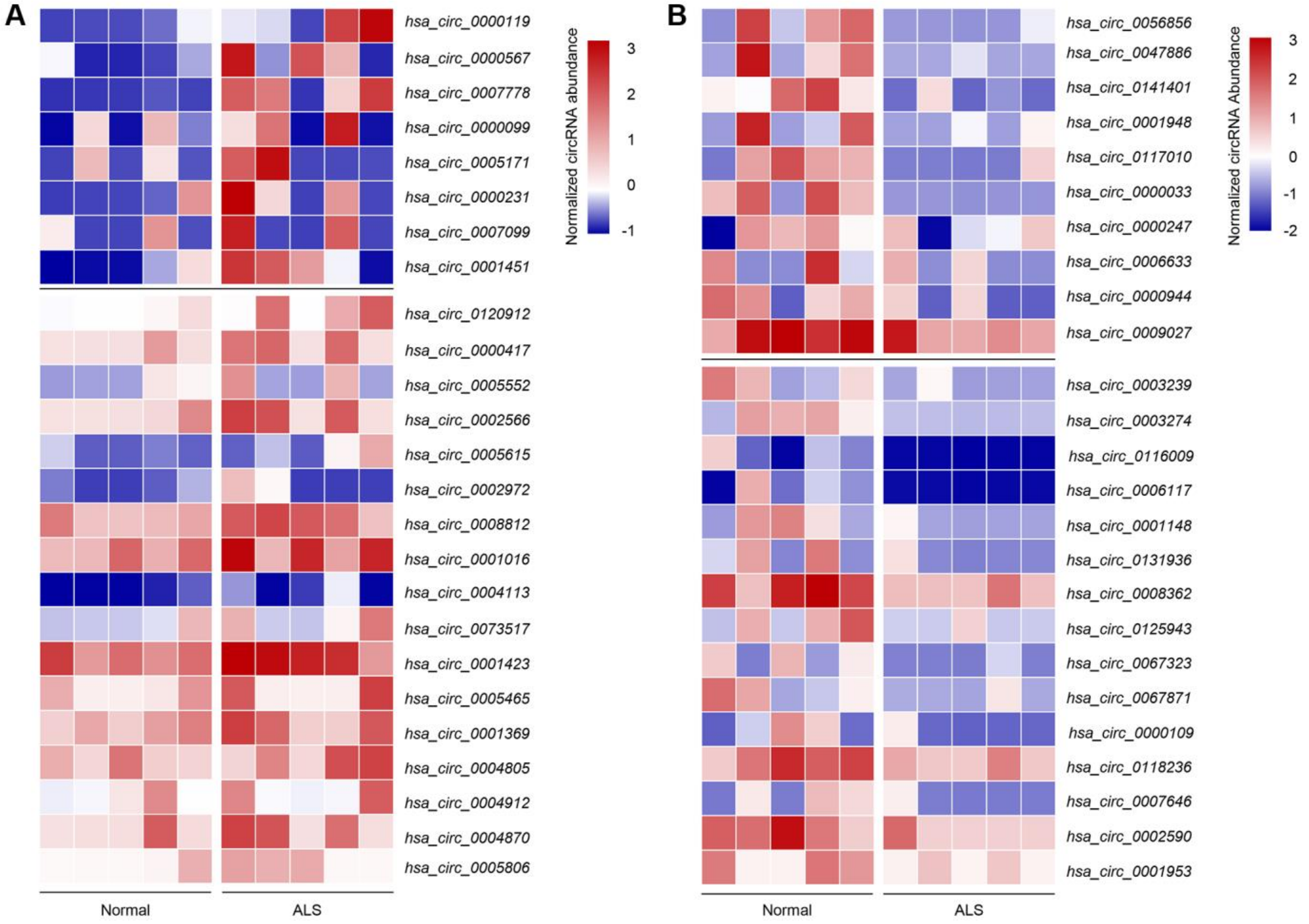


**Supplementary Figure 2. Expression of select circRNAs based on RNA-seq analysis chosen for RT-qPCR analysis.** Heatmap of circRNAs [upregulated (**A**) and downregulated (**B**)] predicted to change based on the fold change of the circRNA-enriched RNA-seq analysis in the ALS cohort. The circRNAs whose fold change was later validated by RT-qPCR analysis (*top* part of heatmaps; Figure 2) are separated with a line from circRNAs whose fold change was not validated by RT-qPCR analysis (*bottom* part of heatmaps; Supplementary Figure 3).

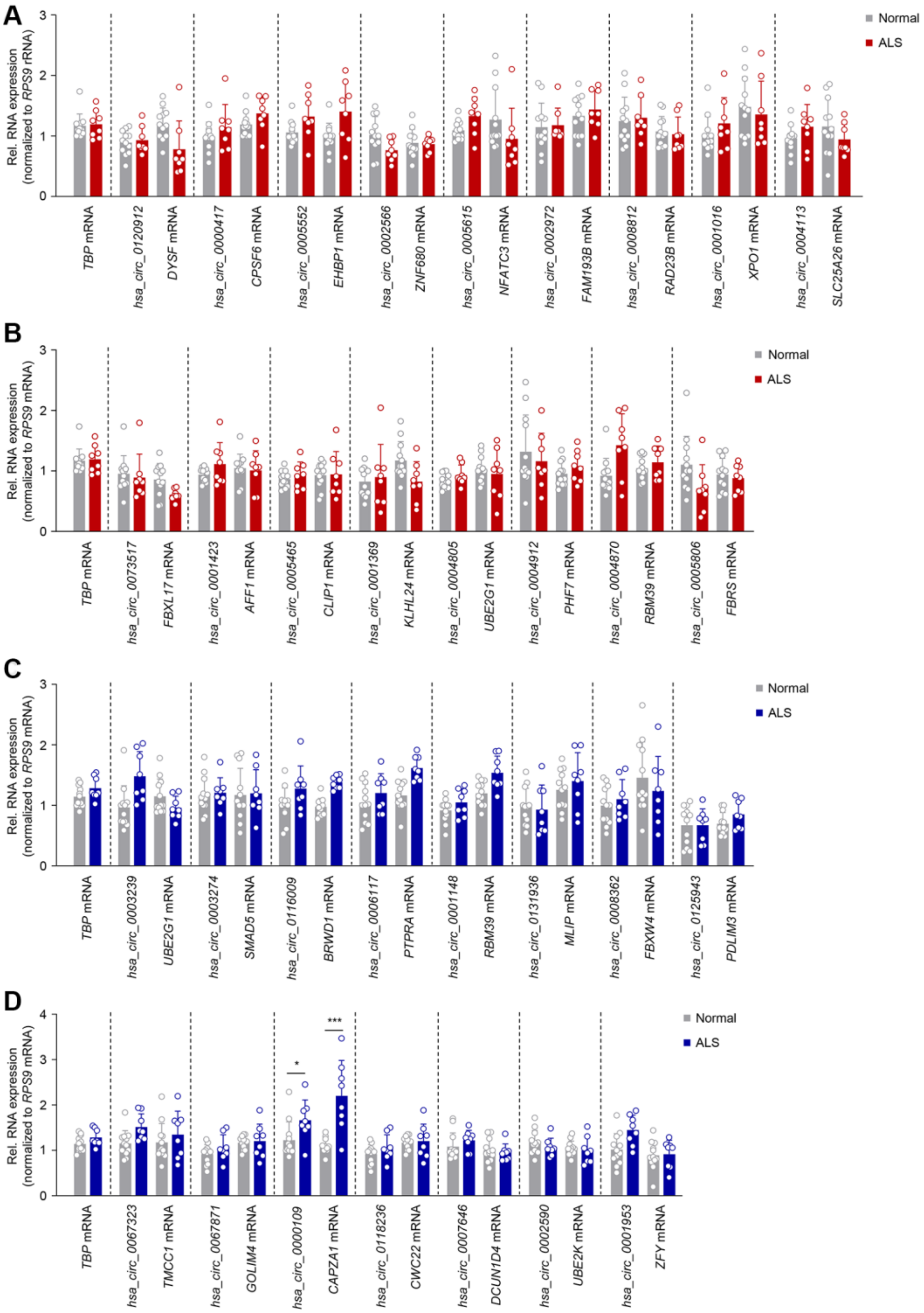


**Supplementary Figure 3. RT-qPCR analysis of circRNAs predicted to change by RNA-seq analysis but not validated.** Differential expression of circRNAs predicted to increase (**A**, **B**) or decrease (**C**, **D**) based on their fold change and their linear counterpart in normal ($n$ = 12) and ALS ($n$ = 8) muscle biopsies, as quantified by RT-qPCR analysis. Data were normalized to *RPS9* mRNA levels, and *TBP* mRNA expression levels were included as a control; $p$-values $^{*}p < 0.05$, $^{***}p < 0.001$.

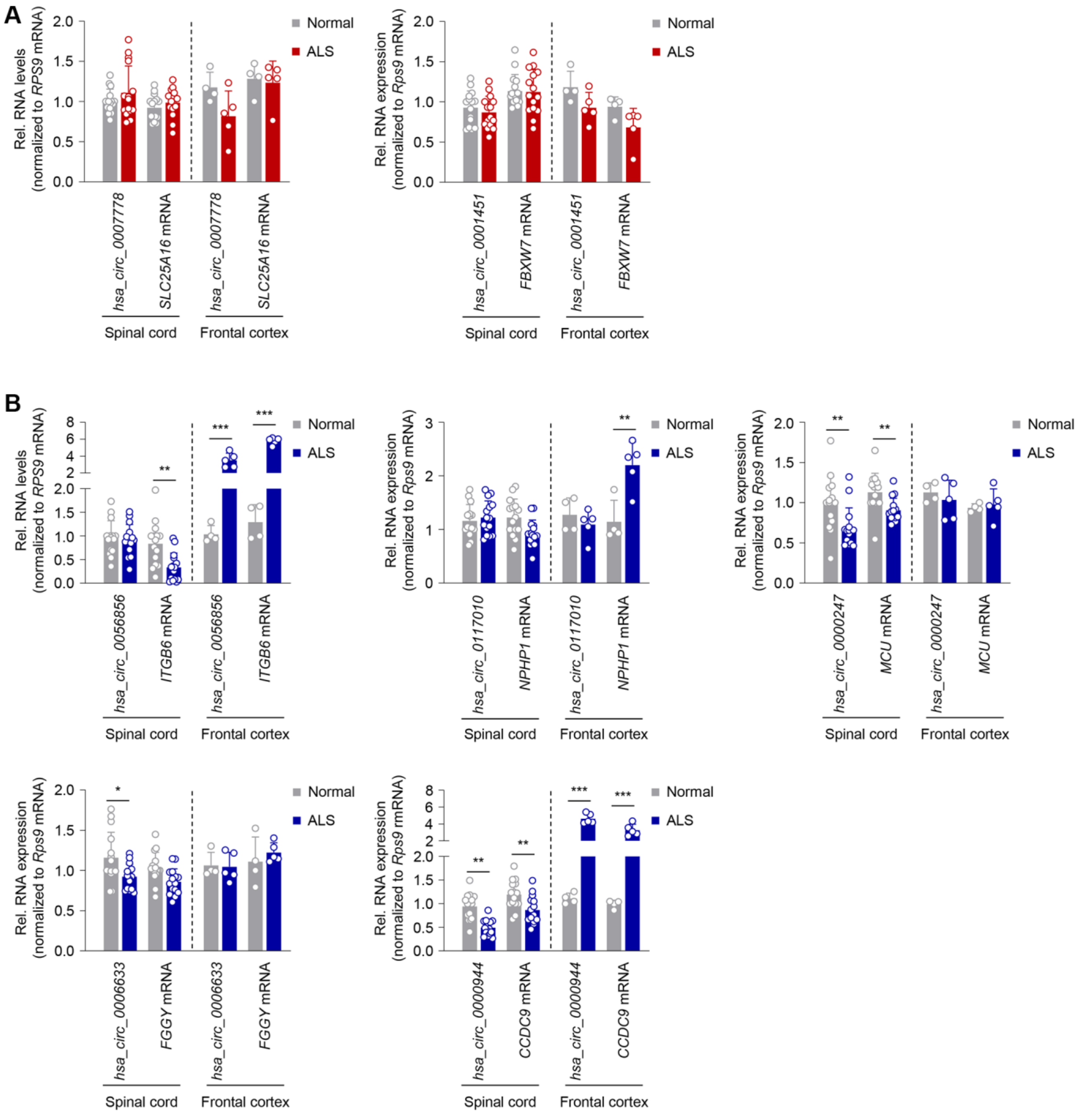


**Supplementary Figure 4. Differential expression in ALS CNS of circRNAs differentially abundant in human ALS muscle.** Differential expression of upregulated (**A**) and downregulated (**B**) circRNAs and their linear counterparts, as validated in human ALS skeletal muscle biopsies, from human spinal cord (cervical, thoracic, lumbar regions; $n$ = 5 for each region in both normal and ALS) and frontal cortex ($n$ = 4 for normal and $n$ = 5 for ALS) biopsies. Data were normalized to *RPS9* mRNA levels; $p$-values $^{*}p < 0.05$, $^{**}p < 0.01$, $^{***}p < 0.001$.

**A**

| circRNA | Host Gene (species) | Exons | Sequence overlap |
|---|---|---|---|
| *hsa_circ_0000119* | NM_006699.5 (*H. sapiens*) | 2-6 | 88% |
| | NM_010763.2 (*M. musculus*) | 2-6 | |
| *hsa_circ_0005171* | NM_018112.3 (*H. sapiens*) | 2-4 | 85% |
| | NM_028053.2 (*M. musculus*) | 2-4 | |
| *hsa_circ_0007099* | NM_152924.5 (*H. sapiens*) | 2-3 | 88% |
| | NM_018811.6 (*M. musculus*) | 2-3 | |
| *hsa_circ_0000567* | NM_032233.3 (*H. sapiens*) | 2-6 | 92% |
| | NM_001364266.1 (*M. musculus*) | 3-7 | |
| *hsa_circ_0000231* | NM_018287.7 (*H. sapiens*) | 2-3 | 87% |
| | NM_001039692.1 (*M. musculus*) | 2-3 | |
| *hsa_circ_0007778* | NM_001324312.2 (*H. sapiens*) | 2-8 | 88% |
| | NM_175194.2 (*M. musculus*) | 2-8 | |
| *hsa_circ_0001451* | NM_033632.3 (*H. sapiens*) | 2-3 | 91% |
| | NM_001177773.1 (*M. musculus*) | 4-5 | |
| *hsa_circ_0000099* | NM_001387437.1 (*H. sapiens*) | 2-9 | 73% |
| | NM_001190403.1 (*M. musculus*) | 2-9 | |

**B**

| circRNA | Host Gene (species) | Exons | Sequence overlap |
|---|---|---|---|
| *hsa_circ_0056856* | NM_000888.5 (*H. sapiens*) | 11-12 | 83% |
| | NM_001159564.1 (*M. musculus*) | 14-15 | |
| *hsa_circ_0000033* | NM_022778.5 (*H. sapiens*) | 4-6 | 85% |
| | NM_144527.3 (*M. musculus*) | 4-6 | |
| *hsa_circ_0047886* | NM_017742.6 (*H. sapiens*) | 4-6 | 86% |
| | NM_001122675.1 (*M. musculus*) | 4-6 | |
| *hsa_circ_0000247* | NM_138357.3 (*H. sapiens*) | 2-3 | 85% |
| | NM_001033259.4 (*M. musculus*) | 2-3 | |
| *hsa_circ_0141401* | NM_052947.4 (*H. sapiens*) | 4 | 70% |
| | NM_001037294.1 (*M. musculus*) | 4 | |
| *hsa_circ_0001948* | NM_001242614.2 (*H. sapiens*) | 4-8 | 69% |
| | NM_001199349.1 (*M. musculus*) | 3-7 | |
| *hsa_circ_0117010* | NM_000272.5 (*H. sapiens*) | 10-16 | 84% |
| | NM_016902.4 (*M. musculus*) | 10-16 | |
| *hsa_circ_0006633* | NM_001113411.2 (*H. sapiens*) | 3-5 | 88% |
| | NM_001113412.1 (*M. musculus*) | 3-5 | |
| *hsa_circ_0000944* | NM_015603.3 (*H. sapiens*) | 6-7 | 86% |
| | NM_001136471.2 (*M. musculus*) | 6-7 | |
| *hsa_circ_0009027* | NM_152493.3 (*H. sapiens*) | 7-8 | 94% |
| | NM_001081098.1 (*M. musculus*) | 7-8 | |

**Supplementary Figure 5. Information on upregulated or downregulated circRNAs overlapping between human and mouse.** Tables summarizing the aliases of human and mouse transcripts, the exons predicted to comprise the circRNA body, and the potential sequence overlap which we used to generate primers spanning the predicted junction; shown are upregulated (**A**) and downregulated (**B**) circRNAs, validated in human ALS muscle by RT-qPCR analysis.

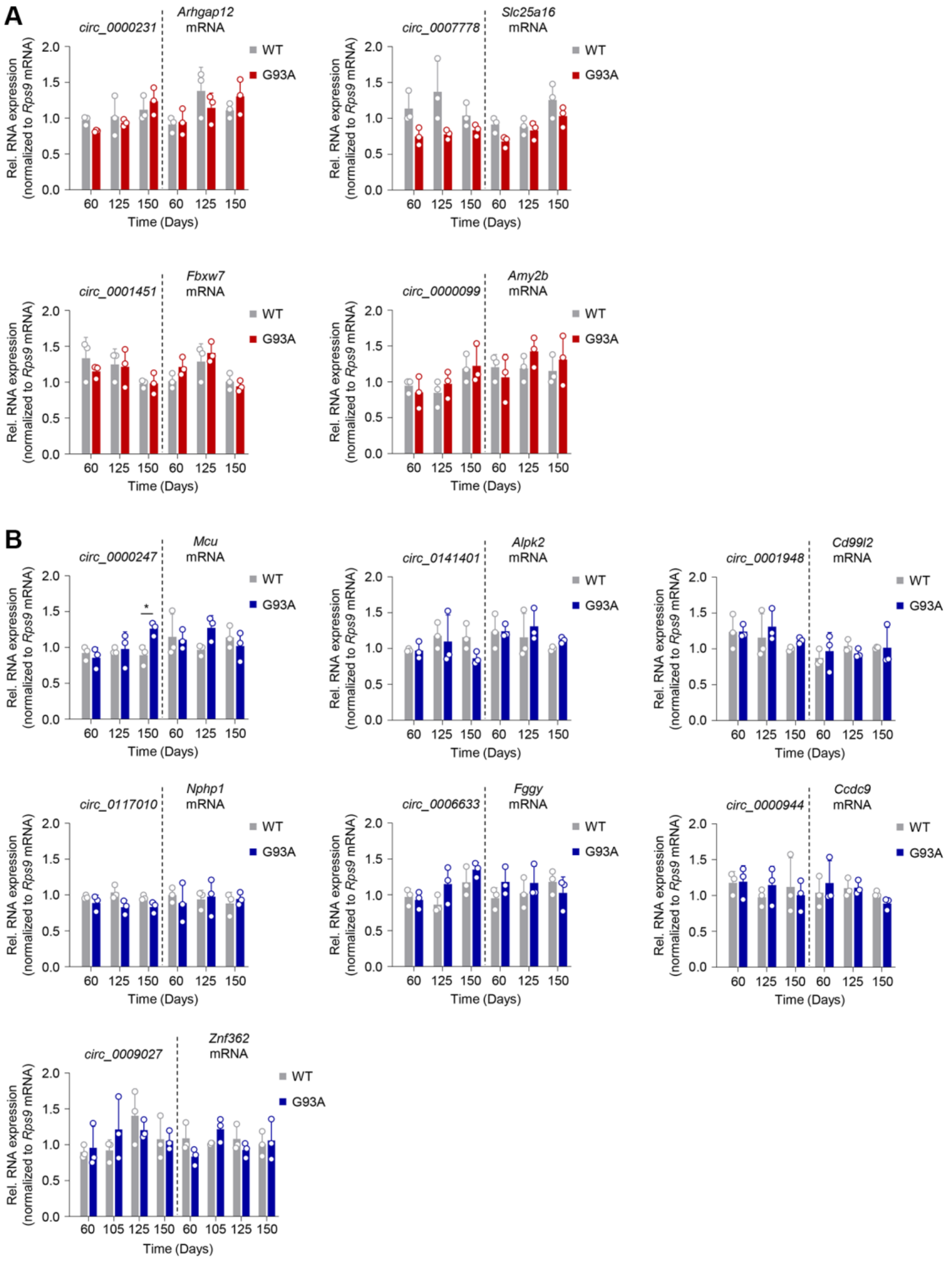


**Supplementary Figure 6. Analysis of expression in muscle of ALS mice (SOD1$^{G93A}$) of circRNAs differentially abundant in human ALS muscle.** CircRNAs that were validated in human ALS muscle and predicted to be upregulated (**A**) or downregulated (**B**), were quantified in skeletal muscle from SOD1$^{G93A}$ mice by RT-qPCR analysis and found to not be significantly altered. Upregulated or downregulated circRNAs are designated by red or blue bars, respectively, based on the validation in human ALS muscle biopsies in Figure 2. Data were normalized to *Rps9* mRNA levels; *p*-values $^{*}p < 0.05$.

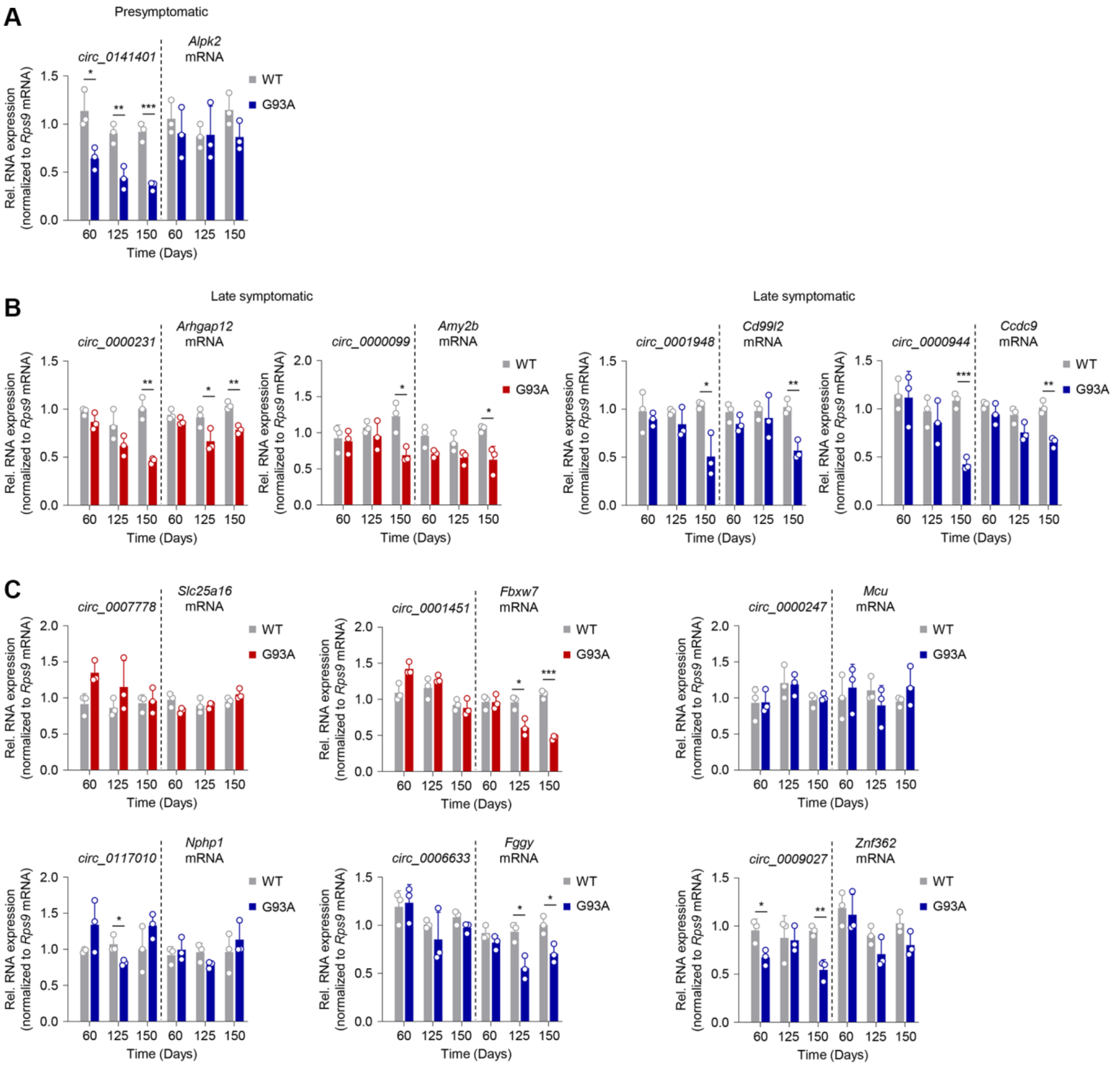


**Supplementary Figure 7. Analysis of expression in spinal cord of ALS mice (SOD1$^{G93A}$) of circRNAs differentially abundant in human ALS muscle.** CircRNAs that were validated in human ALS muscle and predicted to be upregulated (**A**) or downregulated (**B**), were quantified in spinal cord from ALS mice (SOD1$^{G93A}$) by RT-qPCR analysis at the presymptomatic stage (**A**), late symptomatic stage (**B**), and (**C**) not significantly changed. Upregulated (red) or downregulated (blue) designations are based on the circRNA pattern observed in human ALS muscle biopsies in Figure 2. Data were normalized to *Rps9* mRNA; *p*-values $^{*}p < 0.05$, $^{**}p < 0.01$, $^{***}p < 0.001$.
