# Supplementary material for: Transcriptomic analysis of human ALS skeletal muscle reveals a disease-specific pattern of dysregulated circRNAs
Source: Aging (Albany NY). 2022 Dec 30;14(24):9832–59. doi: 10.18632/aging.204450 (PMC9831722; doi:10.18632/aging.204450)
Supplement: Supplementary Table 1 [file aging-14-204450-s005.pdf]

SUPPLEMENTARY TABLE

Supplemental Table 1. Histological features of muscle samples from disease controls.

Neuropathic muscle samples	
**Clinical diagnosis**	**Muscle histology**
Lumbosacral plexopathy	Scattered grouped atrophy; regeneration fibers
CIDP	Type I and II fiber type grouping
Axonal Guillain-Barre syndrome	Angular fibers consistent with mild denervation process
CIDP	Grouped atrophy; angular fibers
Sensorimotor axonal neuropathy	Scattered atrophic fibers; Type I and II fiber type grouping
**Myopathic muscle samples**	
**Diagnosis**	**Muscle histology**
Inflammatory myopathy	Perivascular inflammatory cells and scattered regeneration fibers
Inflammatory myopathy	Perivascular inflammatory cells
Inflammatory myopathy	Endomysial and perivascular inflammatory cells, regeneration fibers
Inflammatory myopathy	Perivascular inflammatory cells
Inflammatory/mitochondrial myopathy	Perivascular inflammatory cells and ragged red fibers
Inclusion body myopathy	Regeneration fibers, rimmed vacuoles, “moth-eaten” fibers
Mitochondrial myopathy	Ragged red fibers, Type II fiber atrophy
Mitochondrial myopathy	Ragged red fibers, Type II fiber atrophy

Abbreviation: CIDP: chronic inflammatory demyelinating polyradiculoneuropathy.
